# Mitochondrial retrograde control of transcription evolves with respiratory stress, metabolic adaptation, and virulence in budding yeasts

**DOI:** 10.1093/molbev/msag005

**Published:** 2026-01-09

**Authors:** Karolina Łabędzka-Dmoch, Thi Hoang Diu Bui, Jakub Piątkowski, Marta Dilling, Paulina Jagiełło, Wiktoria Kabza, Paweł Golik

**Affiliations:** Faculty of Biology, Institute of Genetics and Biotechnology, University of Warsaw, Warsaw, Poland; Faculty of Biology, Institute of Genetics and Biotechnology, University of Warsaw, Warsaw, Poland; Faculty of Biology, Institute of Genetics and Biotechnology, University of Warsaw, Warsaw, Poland; Faculty of Biology, Institute of Genetics and Biotechnology, University of Warsaw, Warsaw, Poland; Polish Academy of Sciences, Institute of Biochemistry and Biophysics, Warsaw, Poland; Faculty of Biology, Institute of Genetics and Biotechnology, University of Warsaw, Warsaw, Poland; Faculty of Biology, Institute of Genetics and Biotechnology, University of Warsaw, Warsaw, Poland; Faculty of Biology, Institute of Genetics and Biotechnology, University of Warsaw, Warsaw, Poland; Polish Academy of Sciences, Institute of Biochemistry and Biophysics, Warsaw, Poland

**Keywords:** retrograde regulation, *Candida albicans*, transcriptional regulation, metabolic adaptation, budding yeasts

## Abstract

The pathway involving the paralogous transcription factors Rtg1 and Rtg3 was first described in *Saccharomyces cerevisiae* as the retrograde regulation that adapts cellular metabolism in response to the state of mitochondrial respiration. We investigated the evolution of this pathway by studying its target genes in respiratory-deficient mutants of *Candida albicans*—a phylogenetically distant and metabolically distinct yeast species. We show that in *C. albicans* the Rtg pathway is also responsible for adaptation to cellular stresses related to respiratory dysfunction, but the repertoire of its target genes is different than in *S. cerevisiae*, and includes genes encoding proteins involved in alternative respiration, oxidative stress, mitophagy, and other aspects of metabolism. We also traced the evolution of the main components of the Rtg pathway and its target genes in the budding yeast (Saccharomycotina) subphylum. We show that the system originated within this clade following a single duplication of the gene encoding the ancestor of Rtg1 and Rtg3, but employs other factors, like the regulatory proteins Rtg2 and Mks1 that were likely present in the last common ancestor of budding yeasts. The regulation of the Rtg transcription factors in *C. albicans* is different than in *S. cerevisiae*, as both Rtg2 and Mks1 were lost in the majority of Serinales. Among the target genes, of particular interest is the evolution of the alternative oxidase (Aox), which was either lost or duplicated in multiple independent events. The presence of Aox strongly correlates with the mitochondrially encoded Complex I—a major source of oxidative stress.

## Graphical Abstract

**Graphical Abstract:**
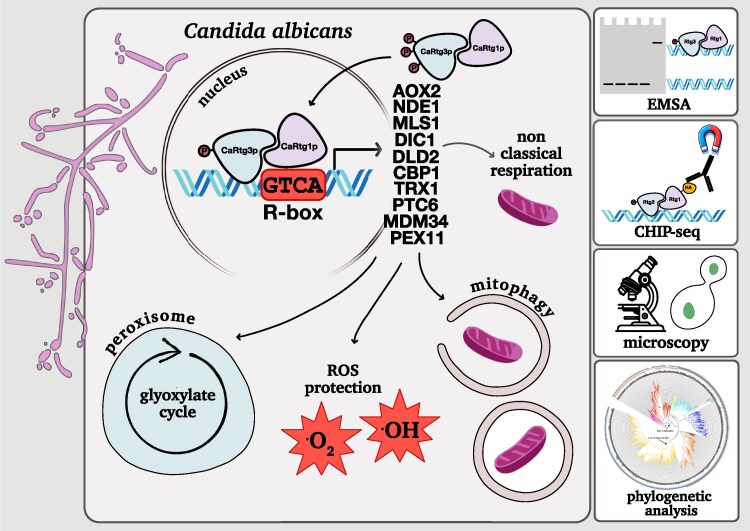

## Introduction

Changes in the regulatory circuits that control gene expression are one of the major drivers of evolution (Carroll 2005; Wray 2007; Tuch et al. 2008; Wohlbach et al. 2009; Thompson et al. 2013; Dalal et al. 2016; Sebé-Pedrós et al. 2016; Johnson 2017). Evolution of genetic signaling and regulatory systems usually proceeds through gene duplication and loss with concomitant processes of neofunctionalization and subfunctionalization leading to functional changes (He and Zhang 2005; Wapinski et al. 2007; Habib et al. 2012). Recent rapid advances in genome sequencing provide ample material for evolutionary genomics, with the budding yeast (subphylum Saccharomycotina within the phylum Ascomycota) being particularly advantageous in such studies (Dujon and Louis 2017; Opulente et al. 2024). However, a well-known feature in the evolution of complex systems is the shift in the function of traits, called exaptation (Gould and Vrba 1982), wherein certain features evolve by natural selection for one function and later are co-opted to perform a different one. On the molecular level, it is manifested as a change in the function of genes and proteins in spite of their orthology. On one hand, it limits the utility of extrapolating the functional studies performed in selected “model” organisms to other species, but on the other hand it can explain the evolution of complex adaptations to different physiological challenges. Comparative experimental studies of functional changes on the molecular level of gene and protein are thus necessary to gain insights into the evolution of cellular and organismal physiology.

One of the defining characteristics of eukaryotic cells is the presence of mitochondria—semi-autonomous organelles of symbiotic origin that retain a vestigial, yet, essential for the respiratory function genome (Gray 2012; Gabaldón 2021; Vosseberg et al. 2024). The two separate, yet functionally interdependent genetic systems have to be coordinated, and the necessary regulatory pathways had to evolve in eukaryotes. Modulation of the nuclear genome expression in response to the physiological state of mitochondria (and chloroplasts), named retrograde regulation (Liu and Butow 2006; de Souza et al. 2017; Bui and Labedzka-Dmoch 2024) is an essential function of all eukaryotic cells. The retrograde (Rtg) signaling pathway was first described in model yeast *Saccharomyces cerevisiae*, where it is responsible for the function of coordinating nuclear and mitochondrial gene expression (Liao and Butow 1993; Chelstowska and Butow 1995; Liu and Butow 2006). Regulatory pathways with a broadly similar function operate in plant (Kleine and Leister 2016; de Souza et al. 2017) and animal (Butow and Avadhani 2004; Jazwinski 2014; English et al. 2020) cells, but the functional similarities do not correspond to evolutionary homology with the yeast Rtg pathway and the system based on the activity of the two Rtg transcription factors (Rtg1 and Rtg3) that was first described in *Saccharomyces cerevisiae* (reviewed in study by Bui and Labedzka-Dmoch [2024]) is specific to the budding yeast subphylum (Saccharomycotina) of the Ascomycetes.

The main players in the *S. cerevisiae* Rtg pathway are two paralogous bHLH transcription factors: Rtg1p and Rtg3p. The Rtg1p/Rtg3p heterodimer binds DNA and activates target gene expression in response to the mitochondrial dysfunction and concomitant decreased glutamate supply (Liu and Butow 1999). Translocation of Rtg1p and Rtg3p to the nucleus, required for the activation of the pathway is regulated by several proteins, the key components being the negative regulator of the pathway—Mks1p, and the positive regulator Rtg2p (Liu et al. 2003). Mks1p promotes the phosphorylation of Rtg3p, which inhibits the nuclear translocation of the Rtg1p/Rtg3p heterodimer. Rtg2p works by binding the negative regulator Mks1p and promoting its dephosphorylation, which in turn keeps it from interacting with Rtg1p and Rtg3p (Liu et al. 2003). The interaction of Rtg2p and Mks1p is promoted by low ATP levels and requires the N-terminal ATP binding domain of Rtg2p, explaining how the mitochondrial dysfunction activates the pathway. Other proteins, such as the positive regulator Grr1p (promoting ubiquitination and subsequent degradation of Mks1p), and negative regulators Bmh1p, Bmh2p, and Lst8p also participate in the regulation of the Rtg pathway, linking it not only to the mitochondrial function but also to the TOR pathway and the levels of glutamate (Liu and Butow 2006). The target genes upregulated by the *S. cerevisiae* Rtg pathway share a common motif in their promoters (the R-box: GTCAC) and include the genes encoding the first four enzymes of the TCA cycle (*CIT1, ACO1, IDH1,* and *IDH2*), as well as the peroxisomal citrate synthase gene *CIT2* (Liu and Butow 2006).

For many decades, *S. cerevisiae* served as the model organism of choice in the study of mitonuclear genetic interactions (Dujon 2020); yet, many aspects of its energy metabolism are relatively recent evolutionary adaptations to the predominantly fermentative lifestyle and are not typical of other yeast lineages (Hagman et al. 2013; Williams et al. 2015; Nespolo et al. 2020). Ascomycetous yeasts are a very diverse and ancient clade with a wide range of different physiological adaptations (Shen et al. 2016; Dujon and Louis 2017; Opulente et al. 2018; Shen et al. 2018; Wolters et al. 2023; Opulente et al. 2024). Whether the composition and function of the retrograde signaling pathway is conserved among various yeast lineages remains an open question due to the paucity of systematic experimental studies in species other than *S. cerevisiae*. The existence of the retrograde signaling pathway involving the Rtg transcription factors in *Komagataella phaffii* (*Pichia pastoris*) was challenged following the observation that the ortholog of Rtg3 in this species lacks conservation of key residues in the putative dimerization (Zip) domain and does not dimerize with Rtg1 (Dey et al. 2018). In *Candida albicans,* the orthologs of Rtg1 and Rtg3 are implicated in the regulation of galactose metabolism genes (*CaGAL1, CaGAL7,* and *CaGAL10*), a function that in *S. cerevisiae* is performed by Gal4p instead (Dalal et al. 2016). However, a later report demonstrated that a Rep1 scaffold coupled with the Cga1 transcription activator is the pivotal regulator of galactose signaling in Serinales, including *C. albicans*, and CaRtg1 and CaRtg3 are required for growth on galactose only upon inhibition of respiration (Sun et al. 2023). CaRtg1 and CaRtg3 were also shown to be involved in sphingolipid homeostasis (Moreno-Velásquez et al. 2020), and adaptation to commensalism and pathogenicity (Pérez et al. 2013). The main regulatory proteins Rtg2 and Mks1 do not have orthologs in *C. albicans* (Liu and Butow 2006; Bui and Labedzka-Dmoch 2024), suggesting that the pathway underwent significant changes in the evolution of different yeast lineages.

On the other hand, the Rtg transcription factors in *C. albicans* control the expression of the inducible alternative oxidase (AOX) gene, which is activated in response to the mitochondrial respiratory dysfunction (Liu et al. 2023; Wenda et al. 2024). This function is important for infectivity, pathogenicity, and survival within the animal host of this yeast species (Pérez et al. 2013; Liu et al. 2023).

The activation of AOX by the Rtg pathway suggests that its function in the retrograde response to mitochondrial dysfunction could be conserved in *C. albicans* despite the considerable evolutionary distance from *S. cerevisiae* and many changes in the components of the pathway (Bui and Labedzka-Dmoch 2024), the mitochondrial genome and respiratory metabolism (Kolondra et al. 2015; Wenda et al. 2024). In this work, we performed a comprehensive study of the target genes activated by the Rtg pathway in respiratory-deficient strains of *C. albicans*, showing that it is indeed involved in the retrograde response to mitochondrial dysfunction, albeit the repertoire of target genes shows only partial overlap with that known in *S. cerevisiae*. To address the ensuing questions concerning the evolutionary origin of the Rtg pathway in budding yeasts and its conservation, we used the unique resource provided by the complete genome sequences of 1,154 yeast strains from 1,051 species that represent nearly all known representatives of this subphylum (Opulente et al. 2024). Applying strict orthology criteria, we show that the paralogous Rtg1 and Rtg3 sequences originated by a single gene duplication early in the evolution of Saccharomycotina from a single ancestral bHLH protein that shows deep homology with proteins from other eukaryotic lineages, including Metazoa. We also traced gene loss events that shaped the unique features of the Rtg pathway in *C. albicans* and related members of the Serinales order and confirmed a very strong correlation between the presence of mitochondrially-encoded Complex I genes and the alternative oxidase.

## Results

### CaRtg1/CaRtg3 transcription factor dimer binds within the proximal promoter of *CaAOX2*

In *C. albicans, CaAOX2* is overexpressed upon electron transport chain (ETC) inhibition (Liu et al. 2023; Wenda et al. 2024). Expression of *CaAOX2* depends on many transcription factors including CaCwt1/CaZcf1, CaZcf2, CaMac1, and CaRtg1/CaRtg3 (Liu et al. 2023). In order to investigate the involvement of CaRtg1/CaRtg3 proteins in the retrograde response to mitochondrial dysfunction, we used the *ΔCaaep3* mutant strain deficient in the biogenesis of mitochondrial ATP synthase that was previously shown to display strong *CaAOX2* induction (Wenda et al. 2024).

**Figure 1:**
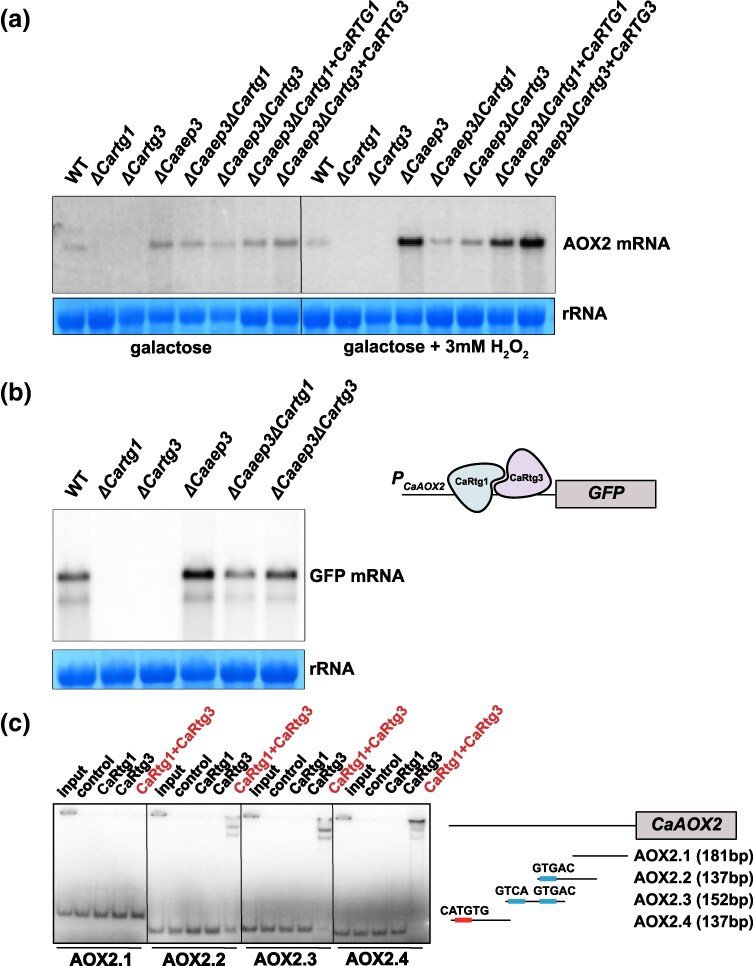
Expression of the *CaAOX2* gene is induced by the CaRtg1/CaRtg3 heterodimer through binding to the *P_CaAOX2_* proximal promoter. (a) Northern blot of total RNA from wild type (WT), deletion mutants, double deletion mutants and reconstitution strains with a restored allele of *CaRTG1* or *CaRTG3* genes grown on galactose with or without the addition of H_2_O_2_ [3 mM] was hybridized with an oligonucleotide probe recognizing the inducible alternative oxidase (*CaAOX2*) mRNA. Methylene blue stained RNA of the large ribosomal subunit is shown as loading control. (b) GFP reporter gene introduced downstream of the *P_CaAOX2_* genomic promoter is induced in wild type (WT), deletion mutants and double deletion strains following the *CaAOX2* expression pattern. Northern blot of total RNA isolated from yeast grown on galactose was hybridized with oligonucleotide probe recognizing GFP mRNA. Methylene blue stained RNA of the large ribosomal subunit is shown as loading control. (c) Electrophoretic mobility shift assay (EMSA) of radioactively labeled DNA probes was performed in 5% non-denaturing polyacrylamide gel with MBP-CaRtg1, MBP-CaRtg3, MBP-CaRtg1+MBP-CaRtg3 or MBP tag or without protein as a control. Canonical GTCA motifs or its derivatives are shown in blue, bHLH binding E-box is shown in red.

We performed northern blot analysis of the steady-state levels of *CaAOX2* mRNA in the *ΔCaaep3* mutant, the *ΔCartg1* and *ΔCartg3* strains, as well as *ΔCaaep3ΔCartg3* and *ΔCaaep3ΔCartg1* double mutants grown on poorly fermentable medium (galactose), also with additional oxidative stress induced by H_2_O_2_. In respiratory competent *ΔCartg1* and *ΔCartg3* strains, we observe a complete loss of *CaAOX2* expression. The induction of its expression in the respiratory-deficient *ΔCaaep3* background, while apparent, is noticeably weaker than in the strains with functional CaRtg1 and CaRtg3 transcription factors (Fig. 1a). This effect is much more pronounced in the cells subjected to additional oxidative stress. This result indicates that CaRtg1/CaRtg3 contribute to the *CaAOX2* induction in respiratory-deficient strains, even though they are not the only regulatory factors involved. The GFP reporter placed under the control of *CaAOX2* promoter (*P_CaAOX2_*) exhibits similar expression pattern to the native *CaAOX2* mRNA in the analyzed mutants (Fig. 1b).

In an earlier study, Liu et al. (2023) demonstrated multiple canonical binding motifs (CARbox) for CaRtg1/CaRtg3 within P*_CaAOX2_* but suggested the existence of an additional binding sequence. We performed electrophoretic mobility shift assay (EMSA) of the proximal P*_CaAOX2_* fragments (−491 to −11) with CaRtg1 and CaRtg3 proteins. We could show that only sequences containing GTCA, GTGAC and a non-canonical CATGTG (E-box) are bound by the CaRtg1/CaRtg3 dimer (Fig. 1c). We thus propose that the additional binding sequence for the CaRtg1/CaRtg3 dimer is located in this proximal promoter region.

### Identification of target genes activated by the Rtg pathway in *C. albicans* in response to mitochondrial respiratory dysfunction using ChIP-seq and verification by EMSA

Earlier studies on the role of the Rtg transcription factors in *C. albicans* (Pérez et al. 2013; Dalal et al. 2016) were performed in strains with wild-type, functional mitochondrial respiration. Our initial experiments confirmed the earlier results of Liu et al. (2023) implicating these transcription factors in the induction of the alternative oxidase (*CaAOX2*) gene expression in response to impaired mitochondrial oxidative phosphorylation. We performed a systematic screen for target gene promoters bound by the CaRtg1 and CaRtg3 transcription factors in mutant strains deficient in different aspects of mitochondrial respiration—the *ΔCappr13* strain with impaired Complex I function, and the *ΔCaaep3* mutant that shows a pleiotropic phenotype related to the defect in the biogenesis of mitochondrial ATP synthase (Wenda et al. 2024).

**Figure 2:**
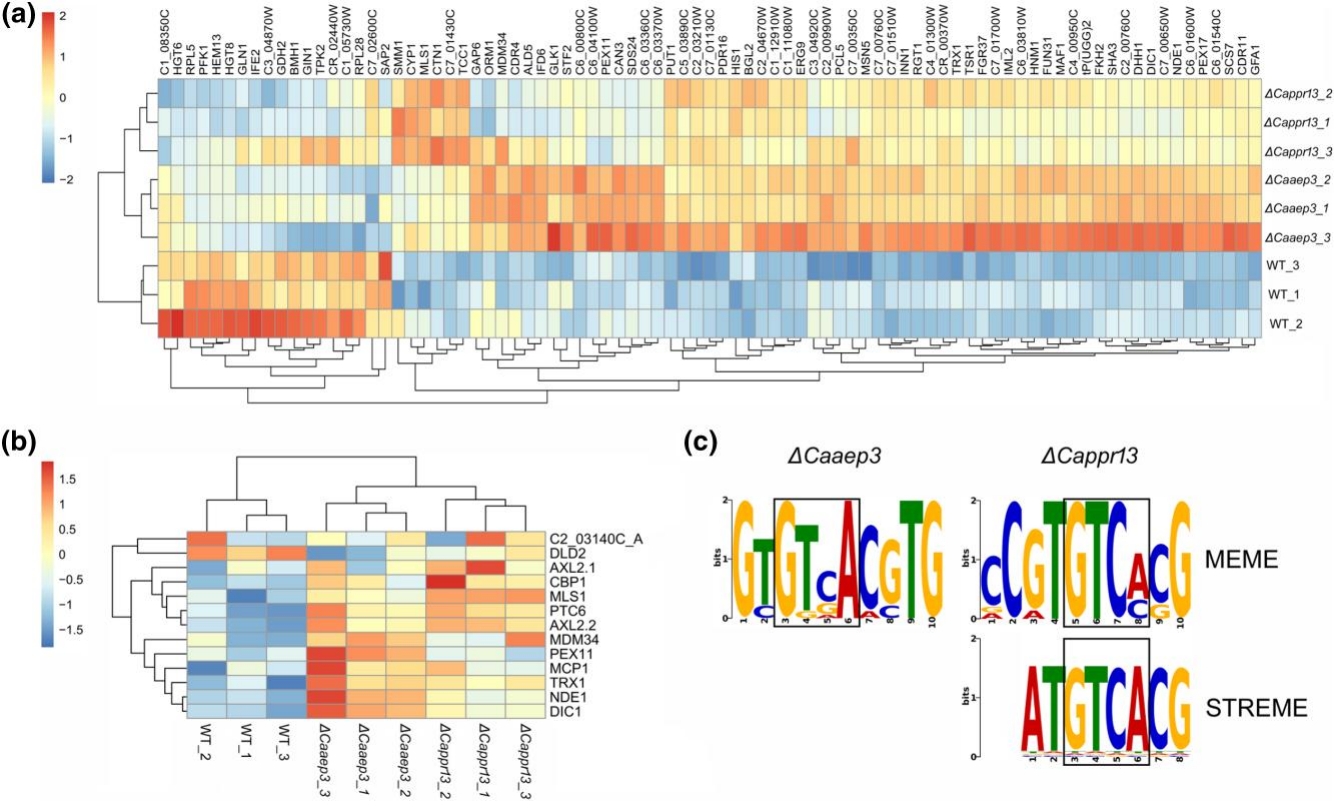
Visualization of ChIP-seq results and motif detection analysis. (a) A heatmap showing genes with significant changes in enrichment in promoter regions for *ΔCaaep3* and *ΔCappr13* (relative to BWP17 WT strain). (b) A heatmap showing changes in enrichment in promoter regions for genes selected for EMSA experiments. (c) LOGO motifs detected with MEME-ChIP (by MEME and STREME algorithms) in sequences significantly enriched in deletion strains.

We performed chromatin immunoprecipitation (ChIP) followed by sequencing of DNA fragments bound by the CaRtg1-HA protein in *ΔCappr13, ΔCaaep3* and wild-type strains, grown on a poorly fermentable carbon source (galactose). We obtained a putative list of the Rtg pathway targets by comparing chromatin fragments enriched in respiratory-deficient strains with the respective set obtained in respiratory proficient control (Fig. 2).

ChIP-seq experiments revealed a high number of differentially enriched promoter sequences in deletion strains. Promoters for 75 genes were significantly differentially enriched in *ΔCaaep3* strain, and for 25 genes in *ΔCappr13* strain, with an overlap of 15 genes (Fig. 2a). A subset of genes thus detected was selected for subsequent EMSA verification (Fig. 2b). Sequences corresponding to differentially enriched peaks were also analyzed with MEME-ChIP in order to detect putative-binding motifs. The CARbox motif was detected in differentially enriched sequences for both *ΔCaaep3* and *ΔCappr13* strains (Fig. 2c). Pathway-enrichment analysis for BWP17 WT strain and deletion strains revealed pathways involved in, among others, nucleophagy, autophagy of mitochondria, and sugar metabolism. However, said results suffer from relatively low confidence levels (Figure S1).

**Figure 3:**
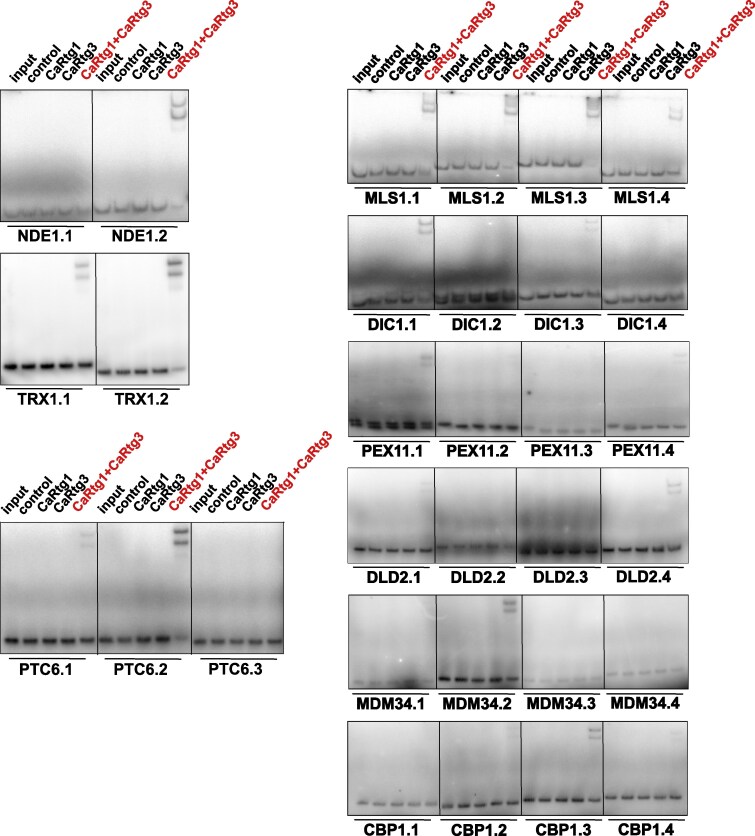
CaRtg1/CaRtg3 heterodimer binds to canonical GTCA motifs in the promoter regions of newly identified target genes. Dimeric MBP-CaRtg1/MBP-CaRtg3 (CaRtg1+CaRtg3), but not monomeric MBP-CaRtg1 (CaRtg1) or MBP-CaRtg3 (CaRtg3) or MBP tag (control) bind to the GTCA containing motifs of the *CaNDE1* (NDE1.2), *CaTRX1* (TRX1.2), *CaPTC6* (PTC6.2), *CaMLS1* (MLS1.1, MLS1.2, MLS1.3), *CaDIC1* (DIC1.1), *CaPEX11* (PEX11.1), *CaDLD2* (DLD2.4), *CaMDM34* (MDM34.2), and *CaCBP1* (CBP1.3) but not to control fragments without the motif (NDE1.1, TRX1.1, PTC6.1, MLS1.4, DIC1.4 PEX11.4, DLD2.3, MDM34.3 and CBP1.4). EMSA of the radioactively labeled DNA probes was performed in 5% non-denaturing polyacrylamide gel with MBP-CaRtg, MBP-CaRtg3, MBP-CaRtg1+MBP-CaRtg3, MBP tag (control) or without protein (input).

From among the newly identified retrograde pathway targets, we selected 12 most interesting candidates for verification by EMSA. We aimed to confirm the binding of the CaRtg1/CaRtg3 transcription factor dimer to the canonical motifs in the promoters of the potential targets. PCR amplified promoter fragments containing GTCA sequences or control fragments without these motifs were chosen to avoid the presence of a TGAC sequence (antiparallel to the GTCA motif from the opposite DNA strand). We confirmed the binding of the CaRtg1/CaRtg3 dimer to the single GTCA motif in the proximal promoter (≤1 kb) of *CaNDE1*, encoding a putative peripheral NADH dehydrogenase, which might serve as an element of the branched respiratory chain (Fig. 3). We also identified three binding motifs in the promoter of *CaMLS1* coding for malate synthase—the enzyme of glyoxylate cycle (Hartig et al. 1992), which is not regulated by the retrograde signaling in *S. cerevisiae* (Fig. 3).

In the ChIP-seq screen, we found three putative target genes related to mitophagy, namely: *CaPTC6*, an ortholog of *ScPTC6* (*AUP1/PPP2*) phosphatase, known to dephosphorylate ScRtg3 (Journo et al. 2009), *CaMDM34* involved in ER and mitochondria tethering via endoplasmic reticulum-mitochondria encounter structures (ERMES) (Böckler and Westermann 2014), and *CaPEX11*—an ortholog of *ScPEX11* involved in the anchoring of peroxisomes with mitochondria (Mattiazzi Ušaj et al. 2015). We could confirm the binding of CaRtg1/CaRtg3 dimer to the GTCA containing fragments of the *CaPTC6* (PTC6.2), *CaMDM34* (MDM34.2) and *CaPEX11* (PEX11.1) promoters (Fig. 3), but not to the fragments devoid of the binding motifs (PTC6.1, MDM34.3 and PEX11.4, respectively).

An interesting retrograde signaling target is encoded by the C6_03390W ORF. We named it *CaDIC1* due to its orthology with *ScDIC1*. The deletion of *S. cerevisiae* ortholog causes vacuole fragmentation and mitochondrial genome loss (Punzi et al. 2018). We were able to confirm specific binding of the CaRtg1/CaRtg3 heterodimer to the fragment of the *CaDIC1* promoter containing a single GTCA fragment (DIC1.1) (Fig. 3).

CaTrx1 (thioredoxin) is a cytoplasmic protein involved in oxidative stress response (da Silva Dantas et al. 2010; Day et al. 2024), making it an attractive candidate as the target of the retrograde signaling. We confirmed the binding of a single GTCA motif from the promoter of *CaTRX1* to the CaRtg1/CaRtg3 transcription factor, with simultaneous reduction of the unbound probe level (TRX1.2), in contrast to the control TRX1.1 fragment, where the gel shift was observed, however, with no detectable decline of the free probe (Fig. 3).

The *CaCBP1* (CR_09270C) gene (not to be confused with the unrelated *CBP1* of *S. cerevisiae*) has no ortholog in the *S. cerevisiae* genome and encodes a protein with a NAD/FAD-binding domain that was shown to be crucial for *C. albicans* virulence and drug resistance (Malloy et al. 1993; Dhamgaye et al. 2012). Its proximal promoter (≤1 kb) possesses seven GTCA motifs and yet, the binding to the CaRtg1/CaRtg3 heterodimer was confirmed for only one fragment carrying a single motif (CBP1.3, Fig. 3).

We also performed EMSA verification with four fragments of *CaDLD2* promoter and confirmed the binding of CaRtg1/CaRtg3 to the fragment possessing a single GTCA motif (DLD2.1) but not to three other fragments (Fig. 3). *CaDLD2* codes for D-lactate dehydrogenase, which localizes to the mitochondrial inner membrane, making it a potentially interesting target of the retrograde response.

We were unable to confirm the binding of CaRtg1/CaRtg3 in the promoters of three genes initially selected for verification: *CaDDL1*, *CaMCP1* and *CaAXL2* (Figure S2).

### Constitutive nuclear localization of the CaRtg1/CaRtg3 dimer depends on the presence of phosphorylated CaRtg3

**Figure 4:**
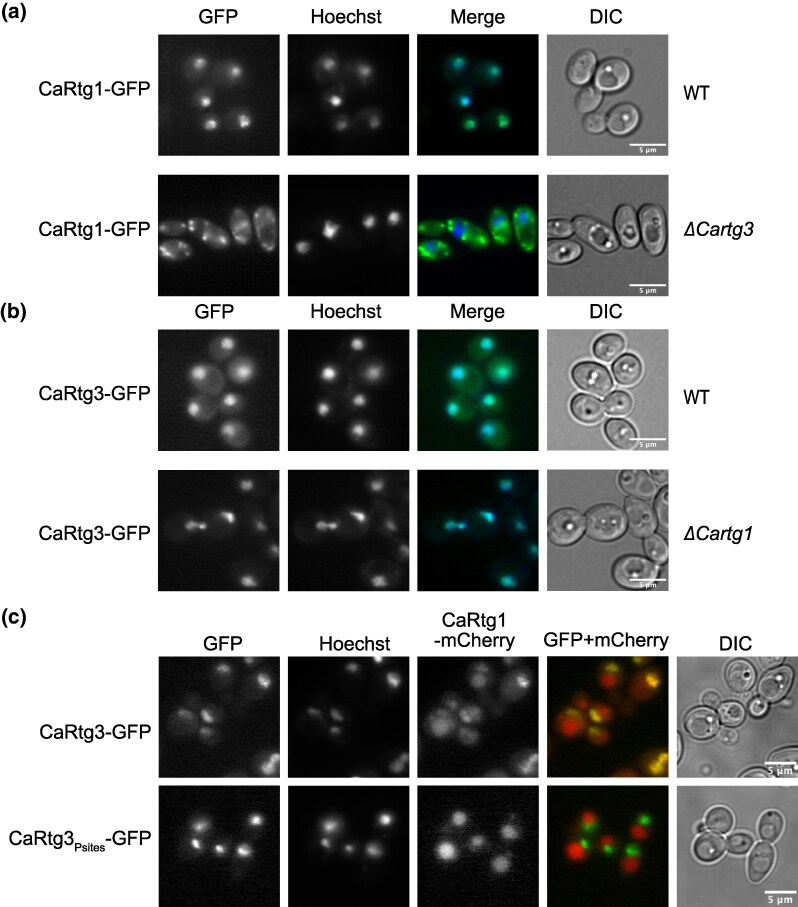
Nuclear localization of CaRtg1 and CaRtg3 depends on CaRtg3 containing six phosphoserines. Fluorescent imaging of CaRtg1-GFP, CaRtg3-GFP in WT, and *ΔCartg1* (a) and *ΔCartg3* (b) deletion strains is shown. (c) Colocalization of CaRtg1-mCherry with CaRtg3-GFP but not CaRtg3_Psites_-GFP (with serines S_231_, S_235_, S_247_, S_251_, S_398_, S_401_ mutated to alanines). Nuclei are labeled with Hoechst33342, DIC (Differential Interference Contrast) is shown as the reference image. The scale bar corresponds to 5 μm.

In *S. cerevisiae,* induction of the retrograde pathway in response to mitochondrial dysfunction depends on translocation of the ScRtg1/ScRtg3 heterodimer to the nucleus (Bui and Labedzka-Dmoch 2024). Conversely, earlier studies suggest that in *C. albicans* the CaRtg1/CaRtg3 heterodimer could be permanently located in the nucleus (Yan et al. 2014; Moreno-Velásquez et al. 2020; Liu et al. 2023). In order to investigate the subcellular localization of the Rtg transcription factors in respiratory-deficient and competent cells, we constructed CaRtg1-GFP and CaRtg3-GFP C-terminal fusions under the control of their native promoters. We found that both proteins localize permanently in the nucleus, regardless of their respiratory phenotype (Fig. 4a and b, Figure S3a and b). CaRtg3 remains in the nucleus in the absence of CaRtg1 (*ΔCartg1* strain). However, in the *ΔCartg3* strain, CaRtg1 shows extranuclear localization regardless of the respiratory capacity (Fig. 4a and b and Figure S3a and b).

High throughput analysis of the *C. albicans* phosphoproteome (Willger et al. 2015) indicated that CaRtg3 is phosphorylated and thus, we decided to investigate whether the phosphorylation state is important for its nuclear localization. We replaced six serine residues, which were shown to be phosphorylated (S_231_, S_235_, S_247_, S_251_, S_398_, S_401_) (Willger et al. 2015), with alanines and tested the localization of the mutein fused with GFP tag (CaRtg3_Psites_-GFP). We found that CaRtg3_Psites_-GFP is able to translocate to the nucleus comparably to the wild type protein. However, when we simultaneously followed the localization of CaRtg1-mCherry, it showed nuclear colocalization with the wild-type CaRtg3-GFP but not with CaRtg3_Psites_-GFP (Fig. 4c), indicating that in *C. albicans* the key phosphoserines are important for the CaRtg3-dependent nuclear localization of both partners.

### The CaRtg1 and CaRtg3 transcription factors could be regulated by modulating their protein levels

In *S. cerevisiae,* in addition to ScRtg1/ScRtg3 heterodimer, there are other cytoplasmatic regulators of the Rtg pathway, most importantly ScRtg2 and ScMks1, which control translocation of the Rtg transcription factors from cytoplasm to the nucleus by a mechanism that involves changes in protein phosphorylation (Bui and Labedzka-Dmoch 2024). Given the constitutive nuclear localization of CaRtg1 and CaRtg3 regardless of the respiratory state, and the absence of orthologs of these two regulatory factors, we suspected that a different mechanism activates the response mediated by the CaRtg1/CaRtg3 dimer in *C. albicans*.

**Figure 5:**
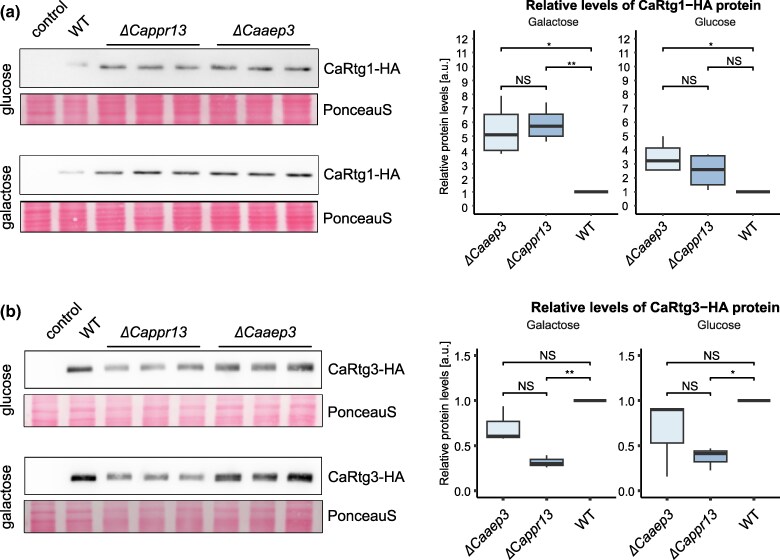
CaRtg1-HA and CaRtg3-HA protein levels change in respiratory-deficient strains. (a) CaRtg1-HA shows significantly higher levels in both respiratory-deficient strains (*ΔCappr13* and *ΔCaaep3*) when compared with the wild type on glucose and galactose. (b) CaRtg3-HA shows decreased expression in *ΔCappr13* but not in *ΔCaaep3* compared to the wild type. Western blot analysis of CaRtg1-HA and CaRtg3-HA in *ΔCappr13, ΔCaaep3* (in triplicates) and wild type strains grown on glucose or galactose respectively was performed with 100 µg of total protein separated by 10% SDS-PAGE gel and immunoblotted with anti-HA antibody. Ponceau S staining was performed for total protein normalization. Averaged WT level was arbitrarily set as 1. Quantitative analysis was performed by FIJI software (Schindelin et al. 2012). Quantification was repeated with 3 independent experiments and analyzed statistically (ANOVA and student's *t*-test); *P*-value—0 < *** < 0.001 < ** < 0.01 < * < 0.05; NS, non-significant.

While performing the ChIP-seq experiment, when we compared the levels of the recovered HA-tagged CaRtg1 protein in *ΔCappr13* and *ΔCaaep3* strains with the wild type, we observed that they vary depending on the respiratory competence. To further elucidate these differences, we performed quantitative analysis of CaRtg1-HA and CaRtg3-HA in the respiratory-deficient and wild-type strains grown on the fermentable (glucose) and poorly fermentable (galactose) carbon sources. Both respiratory-deficient strains display higher CaRtg1-HA levels even on glucose, and the difference is more pronounced on galactose (Fig. 5a). On the other hand, the level of CaRtg3-HA is decreased in *ΔCappr13* strain but not in *ΔCaaep3* when compared to the wild type (Fig. 5b). The effect is, however, less pronounced than in the case of CaRtg1-HA. These results point to a possibility that in *C. albicans*, in the absence of Rtg2 and Mks1, the induction of the retrograde response to respiratory dysfunction might be enacted by modulating the expression levels of the transcription factors themselves, particularly by increasing CaRtg1 expression.

### Evolution of the Rtg signaling pathway in the budding yeast subphylum

#### The retrograde transcription factors Rtg1 and Rtg3 are a result of a single gene duplication event that occurred during the evolution of Saccharomycotina

**Figure 6:**
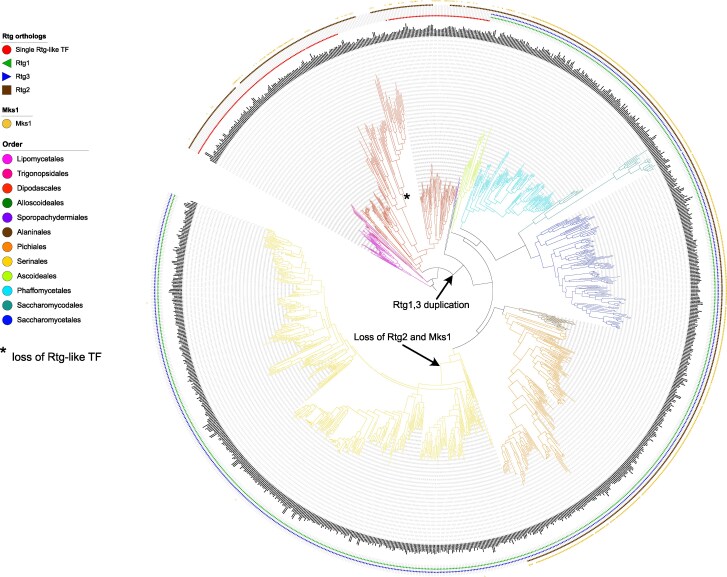
Orthologs of the major components of the retrograde pathway: the Rtg1 and Rtg3 transcription factors (TFs), and the regulatory factors Rtg2 and Mks1 in the phylogenomic tree of 1,154 yeast strains from 1,051 species. The tree and classification into orders were taken from the analysis by Opulente et al. (2024). Filled symbols indicate unambiguous presence of an ortholog, empty symbols—an ortholog with less clear evidence or weaker sequence similarity. Additional details of the analysis not described in the main text, as well as alternative presentation of the data, can be found in the Supplementary material. See the Data Availability Statement for a link to the interactive phylogenetic trees.

In order to investigate the evolutionary origin of the Rtg pathway, we first searched for orthologs of the two transcription factors Rtg1 and Rtg3 in the published genomes of 1,154 yeast strains (Opulente et al. 2024). As the Rtg1 and Rtg3 proteins share significant sequence similarity, they were placed in the same orthology group by the pipelines used by Opulente et al. (2024), and their similarity to other members of the bHLH transcription factor family requires careful application of the strict reciprocal best hit criterion to identify true orthologs of well-known *S. cerevisiae* Rtg1p and Rtg3p sequences that were used as queries. The results (Fig. 6, Figure S4) indicate that orthologs of the Rtg proteins can be found in the majority (1,118/1,154; 98%) of budding yeasts. The absence of any Rtg ortholog is mostly limited to a single clade in the order Dipodascales containing *Starmerella* sp. and four of *Wickerhamiella* sp. (Fig. 6, Figure S4) and probably results from a single gene loss event. The other *Wickerhamiella* sp. do have an Rtg ortholog, albeit the protein is shorter than most orthologs from related yeasts The sequence identified in *St. floris* in this clade is phylogenetically not related to the Rtg orthologs of *Wickerhamiella* sp. or other Dipodascales (Figure S5) and is thus likely to be either an artifact, or a result of horizontal gene transfer.

In members of the three basal orders (using the classification of Saccharomycotina into 12 orders (Groenewald et al. 2023) and the phylogenomic tree from Opulente et al. 2024)—Lipomycetales, Trigonopsidales, and Dipodascales—we identified only a single ortholog of the Rtg proteins, suggesting a duplication that occurred during the evolution of budding yeasts (Fig. 6, Figure S4). All the remaining orders contain orthologs of both Rtg1 and Rtg3. As earlier studies, that focused on the proposed role of the Rtg factors in the regulation of galactose catabolism pathway (Harrison et al. 2022), suggested that both Rtg1 and Rtg3 orthologs can be found in all the budding yeast lineages, including the early branches, we analyzed the sequences from these three orders that showed significant similarity to either Rtg1 or Rtg3 but failed the reciprocal best hit criterion and found that they were more similar to other bHLH transcription factors from *S. cerevisiae*—Cbf1p or Hms1p. This was also the case for the highest scoring Rtg homologs in the *Starmerella* clade.

**Figure 7:**
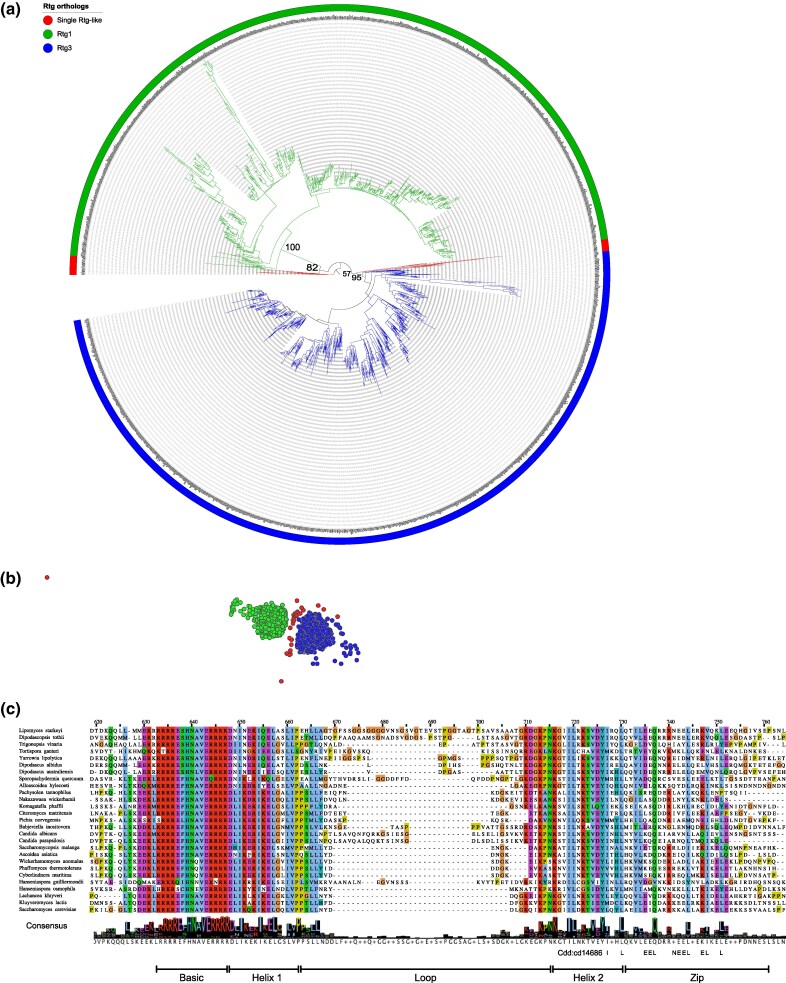
A single duplication resulted in the Rtg1 and Rtg3 paralogous proteins. (a) Maximum-likelihood tree of the Rtg orthologs. Numbers indicate bootstrap values for selected nodes. The orthologs of either Rtg1 or Rtg3 of *S. cerevisiae* (2017 sequences) were filtered to remove incomplete and/or concatenated sequences, resulting in a set of 1,804 sequences that were aligned. The alignment was trimmed to remove noninformative regions (leaving 115 aa remaining) and the consensus ML tree of 1,000 bootstrap replicates was obtained as described in Materials and Methods. The tree was rooted using Lipomycetales as outgroup (the unrooted tree is available as Figure S11). (b) Clustering of the Rtg orthologs based on pairwise similarity (estimated by BLAST) using the Fruchterman–Reingold graph layout algorithm in the 3D space in CLANS (Frickey and Lupas 2004). The colors marking Rtg1, Rtg3, and single Rtg-like sequences are as in (a). (c) Multiple sequence alignment of 27 representative Rtg3 sequences. The regions are indicated according to the earlier study in *S. cerevisiae* (Jia et al. 1997). Selected residues from the consensus of the Zip region are based on entry cd14686 from the Conserved Domain Database (Wang et al. 2023). See the Data Availability Statement for a link to the interactive phylogenetic trees.

To further confirm that a single duplication event during the evolution of Saccharomycotina resulted in the paralogous Rtg1 and Rtg3 transcription factors, we performed phylogenetic analysis of 1,804 amino acid sequences of Rtg orthologs. The results (Fig. 7a) clearly show that the Rtg1 orthologs and Rtg3 orthologs group together as single well-supported clades (bootstrap values 100 and 95 for the Rtg1 and Rtg3 groupings, respectively), among more divergent Rtg-like sequences from the three basal orders. Similar results were obtained using CLANS 2 clustering of the Rtg sequences (Fig. 7b).

Earlier study (Dey et al. 2018) of the Rtg proteins from *Komagataella phaffii* (*Pichia pastoris*) suggested that the Rtg3 ortholog in this species lacks conservation of residues identified as parts of the leucine zipper (Zip) dimerization domain in *S. cerevisiae* (Jia et al. 1997). In order to investigate the conservation of Rtg3 functional domains in a broader phylogenomic context, we performed a multiple alignment of 27 Rtg3 orthologs from species representing all orders of Saccharomycotina. The results (Fig. 7c) show that the bHLH transcription factor motifs (basic, helix 1 and helix 2) are well conserved, with the loop region showing more variability in length and sequence. Importantly, the dimerization (Zip) region is also generally conserved, and differences from the consensus (cd14686) from the Conserved Domain Database (Wang et al. 2023) are apparent also in species such as *S. cerevisiae* and *C. albicans*. This suggests that all the Rtg3 orthologs (as well as single Rtgs in the basal orders) are likely to function as bHLH transcription factors, although their capability to form dimers cannot be reliably determined based on sequence alone. In particular, even though the protein from *K. phaffi* was shown to be incapable of forming heterodimers with the Rtg1 ortholog in vitro (Dey et al. 2018), based on the sequence conservation it should nevertheless be considered as an Rtg3 ortholog despite potential changes in function.

As to further investigate the origins of the Rtg regulatory pathway, we looked for orthologs of Rtg1 and Rtg3 in representative genomes of other Fungi and more distant taxa (Figure S6). Sequences fulfilling the reciprocal best hit criterion for orthology to Rtg3p of *S. cerevisiae* can be found not only in other Fungi (with the notable exception of *Schizosaccharomyces pombe*), but also in other eukaryotic lineages, including Metazoa. The closest human homolog of Rtg3p is an isoform of the microphthalmia-associated transcription factor (MITF) (Goding and Arnheiter 2019). Orthologs of Rtg1p, on the other hand, seem to be limited to budding yeasts, suggesting that the ancestral Rtg transcription factor was closer to Rtg3. This ancestral gene underwent a single duplication event within Saccharomycotina after the separation of the earliest lineages (Lipomycetales, Trigonopsidales, and Dipodascales) from the other taxa, although the exact relationships of the well supported Rtg1 and Rtg3 clades to the single Rtg proteins cannot be reliably inferred from the tree.

#### The regulatory factors of the retrograde pathway—Rtg2 and Mks1—are ancient proteins that were lost during the evolution of Serinales

Other major elements of the retrograde signaling system, as known from *S. cerevisiae*, are the two regulatory factors: Rtg2 and Mks1. Orthologs of both these proteins are, however, conspicuously absent from *C. albicans*. Searching the budding yeast genome sequences, we found that Rtg2 and, to a lesser extent, Mks1 are found in species belonging to all of the budding yeast orders, including the basal ones (Fig. 6, Figure S4). The loss of both these proteins occurred during the evolution of the Serinales clade that includes such well-known yeasts as *C. albicans* and *C. parapsilosis*. Six members of this order, forming the earliest branching clade in the tree (*Babjeviella inositovora, Candida chilensis, Candida pseudocylindracea, Cephaloascus albidus, Cephaloascus fragrans* and *Limtongozyma cylindracea*) still have Rtg2 and Mks1 orthologs that are absent in all the other members of this clade (Fig. 6, Figure S4). Other species that lack Rtg2 can be found scattered in the order Dipodascales and include some (but not all) of the *Starmerella* species that also lost an Rtg transcription factor ortholog (Fig. 6, Figure S4).

The conservation of Mks1 homologs is weaker, but putative orthologs of this protein can be found in some members of Lipomycetales and many Dipodascales, as well as in most of the species belonging to orders that have duplicated Rtg1 and Rtg3 (with the exception of the majority of Serinales, as mentioned above), suggesting that this protein is ancestral in the budding yeast subphylum.

Sequences orthologous to Rtg2 can also be found in the genomes of other Ascomycota, but only in the subdivision Pezizomycotina, whereas no clear orthologs of Mks1 could be identified outside the budding yeast clade (Figure S6). We also identified a partial protein sequence with strong homology to *S. cerevisiae* Rtg2p encoded in the draft genome of *Mucor lusitanicus*. Overall, our analysis suggests that the ancestors of the regulatory proteins Rtg2 and Mks1 were present in the budding yeast subphylum (and, in the case of Rtg2, also in other Fungi) before the gene duplication event that resulted in the Rtg1 and Rtg3 transcription factors, and both were lost simultaneously in a single event during the evolution of Serinales. In order to further analyze the evolution of the retrograde pathway, we performed phylogenetic studies involving genes that were either previously known to be regulated by the Rtg factors or identified in the course of this study.

#### The alternative oxidase was lost and duplicated multiple times in the evolution of Saccharomycotina

The Rtg pathway and its targets were first described in *S. cerevisiae,* which is in many ways a specialized species. Among the features of *S. cerevisiae,* that are not shared by many other budding yeasts, is the lack of both the mitochondrially encoded Complex I subunits, and the alternative oxidase (AOX) enzyme, which was recently shown to be regulated by the Rtg pathway in *C. albicans* ((Liu et al. 2023) and this work). The loss of mitochondrial Complex I occurred several times in evolution (Schikora-Tamarit et al. 2021; Wolters et al. 2023), most remarkably in the entire clade containing the Saccharomycetales and Saccharomycodales orders. A correlation of the loss of the mitochondrial Complex I genes and the loss of AOX was postulated previously and attributed to differences in the oxidative stress response mechanisms (Schikora-Tamarit et al. 2021).

**Figure 8:**
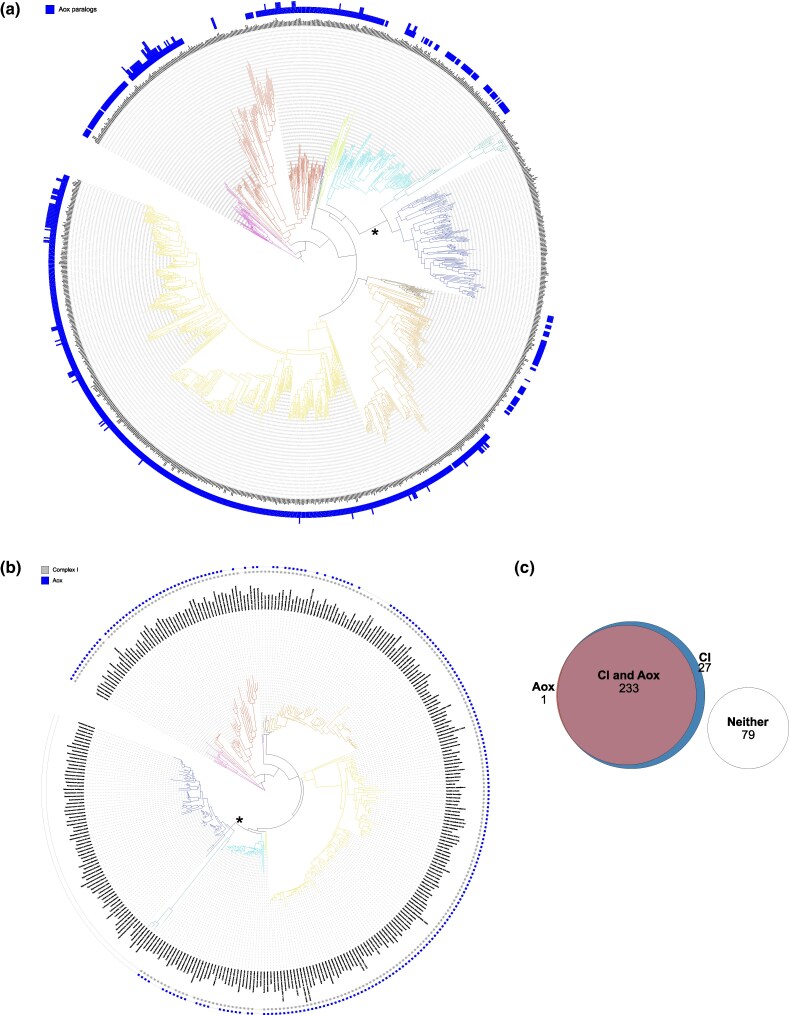
(a) Orthologs of the alternative oxidase (AOX) in the phylogenomic tree of 1,154 yeast strains from 1,051 species. The tree and classification into orders were taken from the analysis by Opulente et al. (2024). The height of the bar corresponds to the number of paralogous Aox sequences in a genome (0 to 4). (b) Presence of the mitochondrial Complex I (CI) genes in mtDNA and the alternative oxidase in the mtDNA tree of 340 strains from Wolters et al. (2023). The asterisk in (a) and (b) marks the major loss of both CI and Aox sequences in the ancestor of Sacharomycetales and Saccharomycodales. The orders in (a) and (b) are colored as in Fig. 6. (c) Venn diagram showing that the presence of mitochondrial Complex I and Aox shows high correlation. Additional details of the analysis not described in the main text, as well as alternative presentation of the data, can be found in the Supplementary material. See the Data Availability Statement for a link to the interactive phylogenetic trees.

In order to investigate the evolution of AOX in budding yeasts, we used the strict reciprocal best hit criterion for orthology with either *C. albicans* Aox1p or Aox2p to search the published budding yeast genome sequences (Opulente et al. 2024). The results (Fig. 8a, Figure S7) indicate that AOX is a common feature among Saccharomycotina (840/1,154, 73%) and was likely present in the last common ancestor of budding yeasts. It was lost in the common ancestor of Sacharomycetales and Saccharomycodales and several independent loss events led to the lack of AOX in some members of other clades, particularly in Phaffomycetales, Dipodascales, and Pichiales, with AOX orthologs in 58%, 71%, and 74% of the species, respectively. On the other hand, the alternative oxidase is present in 100% of Alloscoideatales, Serinales, Sporopachydermiales, and Trigonopsidales.

To investigate the correlation between the presence of AOX and mitochondrial Complex I, we used a subset of 340 yeast strains from the entire dataset of 1,154, which have good quality assemblies of mitochondrial genomes available (Wolters et al. 2023), of which 260 have at least five out of the seven known mitochondrial *NAD* genes. Our results confirmed the correlation between the presence of these features (Fig. 8b and c): 233 species contain both Complex I and AOX, 79 contain neither, 27 species (including some well-known members of Pichiales, such as *Ogataea polymorpha*) have Complex I but lack AOX, and a single species (*Cyberlindnera petersonii*) has an AOX ortholog, but no identified Complex I.

The genome of *C. albicans* encodes not one, but two paralogous alternative oxidases—the constitutive Aox1, and the inducible Aox2 (Huh and Kang 2001). In the majority of 840 yeast species that have an alternative oxidase ortholog, we found only a single Aox sequence. Only in 106 species, we found evidence of paralogous Aox sequences (Fig. 8a, Figure S7), in most cases of two copies. Only among Dipodascales, we found four species with three Aox paralogs and two species with four. Species with single or multiple paralogous Aox sequences are scattered among different orders, suggesting that unlike in the case of the Rtg transcription factors, multiple independent gene duplication and gene loss events occurred in the evolution of budding yeasts.

**Figure 9:**
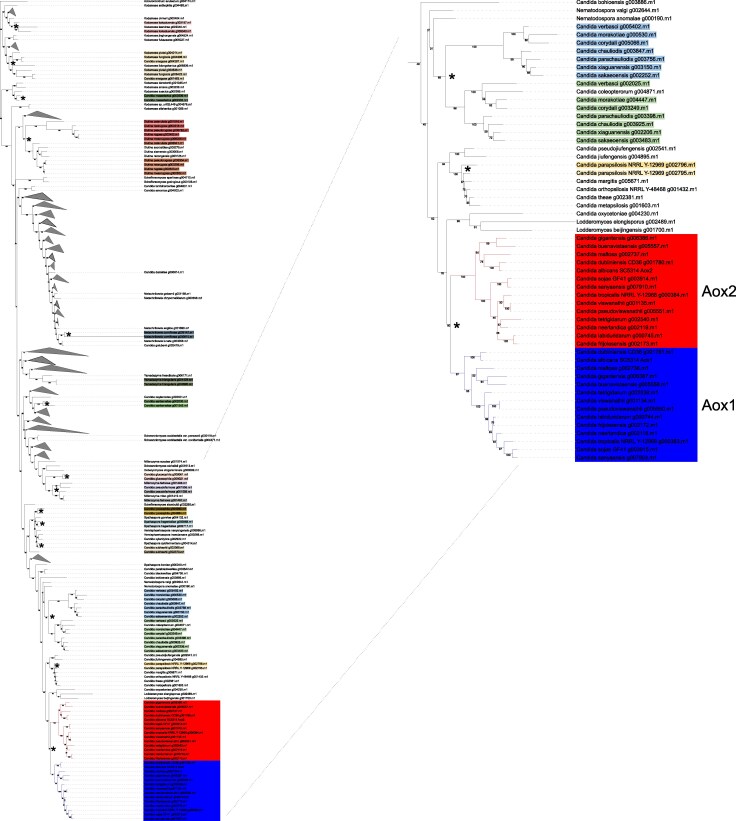
Independent gene duplications in the evolution of the alternative oxidase genes in Serinales. Maximum-likelihood tree of Aox homologs from the order Serinales, with the bootstrap values indicated. The fragment containing *C. albicans*, *C. parapsilosis*, and related species was enlarged. Clades containing exclusively species with a single Aox sequence were collapsed, the uncollapsed tree is available as Figure S8. Species containing paralogous alternative oxidase sequences were marked in color, blue and red background indicates groups of sequences orthologous to *C. albicans* Aox1 and Aox2, respectively. Asterisks mark putative Aox gene duplication events. See the Data Availability Statement for a link to the interactive phylogenetic trees.

In order to verify this hypothesis, we performed phylogenetic analysis of Aox protein sequences in the order Serinales, where each of 430 analyzed species has one or two Aox homologs. The results (Fig. 9, Figure S8) suggest that as many as 13 independent duplications of the alternative oxidase gene occurred during the evolution of this order. One such event occurred in the ancestor of 14 *Candida* species, including *C. albicans*, resulting in sequences orthologous to Aox1 and Aox2 (Fig. 9). The two paralogous Aox sequences of *C. parapsilosis,* on the other hand, cluster together with the single Aox of *C. orthopsilosis*, *C. metapsilosis*, and several other species, suggesting their origin in a separate duplication (Fig. 9). Another separate Aox duplication event occurred in the last common ancestor of seven other *Candida* species, including *C. corydali* (Fig. 9, Figure S8). Other duplications occurred in five *Diutina* species (Fig. 9, Figure S8), and in many other nodes of the Serinales tree. Independent duplications also occurred in other budding yeast orders, for example, in Dipodascales in several *Yarrowia* species, including *Y. lipolytica* (Fig. 8a, Figure S7). Overall, it appears that the single ancestral alternative oxidase of budding yeasts was either lost or duplicated multiple times during the evolution of Saccharomycotina, suggesting a strong selection on its presence and copy number.

#### The canonical targets of the Rtg pathway in *S. cerevisiae* are ancestral and mostly conserved genes with a complex history of paralog expansion and loss

Subsequently, we analyzed the evolution of genes known as canonical targets of the Rtg pathway in *S. cerevisiae,* encoding metabolic enzymes of the TCA cycle (*CIT1, ACO1, IDH1/2*), glyoxylate cycle (*CIT2*) and other pathways (*DLD3* encoding 2-hydroxyglutarate transhydrogenase, or *PYC1* encoding pyruvate carboxylase) (Bui and Labedzka-Dmoch 2024). Each of the 1,154 analyzed budding yeast genomes encodes at least one homolog of these housekeeping enzymes, but their evolutionary history is sometimes complex, particularly in the case of the citrate synthase enzymes.

In all the analyzed genomes, there is at least one homolog of the very highly conserved aconitase genes, with the vast majority (1,072 strains) encoding two paralogs, corresponding to *ACO1* and *ACO2* of *S. cerevisiae*, and 78 genomes possibly containing additional copies (Figure S9). Similarly, the presence of *IDH1* (1,132/1,154 genomes) and *IDH2* (1,151/1,154 genomes) is conserved and ancestral. The vast majority (1,071/1,154) of budding yeasts have two Idh paralogs, and the *IDH* genes underwent additional duplications in a few clades, most notably in *Dipodascus* sp. and in some Saccharomycetales including nine *Tetrapispora* species (Figure S9).

*PYC1* is an ohnolog of the universally conserved *PYC2*, resulting from the whole genome duplication (WGD) event that occurred during the evolution of Saccharomycetaceae (Wolfe and Shields 1997; Byrne and Wolfe 2005), and the presence of two *PYC* paralogs is clearly a feature of post-WGD Saccharomycetaceae, although some of them lost one of the paralogs (Figure S9). The majority of budding yeasts (1,051/1,154) have a single Pyc protein. Putative duplications of the *PYC* gene can also be found in other clades (Figure S9), including some *Alloascoidea* sp., *Wickerhamomyces* sp., and *Dipodascus* sp. The genomes of Alloascoidales are particularly rich in paralogous sequences of the proteins we studied, suggesting genomic duplication events.

Presence of two or three paralogous D-lactate dehydrogenase (Dld) proteins is also nearly universal (a single Dld was found only in 14 genomes in Saccharomycetales and Dipodascales), and likely ancestral, with subsequent duplications increasing the number of Dld paralogs to as many as 10, with the majority of species encoding three (423 genomes) or four (378 genomes) paralogs (Figure S9).

*CIT1* and *CIT2*, encoding the mitochondrial and peroxisomal citrate synthase in *S. cerevisiae*, respectively, are considered to be ohnologs. The presence of three paralogous Cit proteins is not, however, shared by all the post-WGD Saccharomycetaceae (Figure S9). The third paralog, encoded by *CIT3* (methylcitrate/citrate synthase) is not controlled by the Rtg pathway. They share very high sequence similarity, making unambiguous assignment to orthology groups challenging. Among the 1,154 analyzed genomes, 650 contain a single Cit protein, 418 contain two and 73 contain three (Figure S9). In 12 genomes, we found four or more Cit homologs, and the only genome where no citrate synthase gene could be identified belongs to *Hanseniaspora jakobsenii*—a member of a clade known for multiple gene losses and high divergence (Steenwyk et al. 2019; Gonçalves et al. 2025). Lack of this gene in only a single species could also, obviously, be a result of an incomplete genomic sequence (no Cit homolog was found in either of the two *H. jakobsenii* genome assemblies currently available in the NCBI database).

Two or three Cit paralogs are present in the genomes of all Lipomycetales and Trigonopsidales, suggesting that it is an ancestral trait for the budding yeast subphylum. On the other hand, the majority (393) of Serinales, including *C. albicans*, have only a single Cit homolog (with the basal *Babjeviella inositovora*, *Limtongozyma cylindracea,* and *C. pseudocylindracea* being interesting possible exceptions). Similarly, only a single citrate synthase is prevalent among Pichiales. Overall, it seems that the presence of at least two paralogous citrate synthase sequences is an ancestral trait in budding yeasts, and they underwent multiple gene loss and duplication events in the evolution of the subphylum.

#### Genes controlled by the Rtg pathway in *C. albicans* are conserved in the majority of Saccharomycotina

Finally, we investigated the evolution of several genes that were newly identified in this work as targets of Rtg transcriptional regulation in *C. albicans*. Searching for their orthologs in the 1,154 sequenced budding yeast genomes revealed (Figure S10) that they are all conserved in most orders of Saccharomycotina and, based on their presence in the early branching clades, likely ancestral to this subphylum.

Orthologs of *MLS1* (C1_09690W), *NDE1* (C3_03420C), *PTC6* (CR_07770C), *MDM34* (C1_06230C), *AXL2* (C4_04170C), and *DDL1* (C2_03140C) can be found in almost all sequenced strains (1,153, 1,153, 1,148, 1,138, 1,133, and 1,150 out of 1,154, respectively). *PTC6* belongs to a group of paralogous sequences (including also *PTC1, PTC2, PTC4*, and others), with the majority of analyzed strains encoding 3–5 members. In 675 strains *NDE1* has one (*NDE2*, as in the case of *C. albicans*) or two (*NDE2* and *NDI1*, as in the case of *S. cerevisiae*) paralogs. On the other hand, *MDM34* does not show evidence of paralogous duplications in budding yeasts, and duplications of *DDL1* in 254 genomes were mostly confined to orders Dipodascales and Lipomycetales.

*PEX11* (C6_04310W) is almost universally conserved (1,129 out of 1,154 genomes). It was, however, clearly lost in 25 species belonging to the fast-evolving lineage of *Hanseniaspora* sp. Saccharomycodales, known for multiple gene losses (Steenwyk et al. 2019). Similarly, *MCP1* (C7_01750W) is mostly conserved (1,070/1,154 genomes), and was also lost in the fast-evolving Saccharomycodales, and additionally in a clade containing 43 *Kazachstania* sp. in Saccharomycetales. *TRX1* (CR_10350C) is also found in the majority of genomes (1,123/1,154), and its loss is mostly confined to a single group of Dipodascales that includes some *Dipodascus* sp. and *Geotrichum* sp. (Figure S10).

Orthologs of *DIC1* (C6_03390W) are also present in the majority of budding yeasts (935/1154), and yet, they were lost, remarkably, in the entire orders Ascoideales and Saccharomycodales, a group of *Lachancea* sp. and *Kluyveromyces* sp. in Saccharomycetales, a large clade of Pichiales, including, among others, *Pichia* sp. and *Saturnispora* sp., as well as a few *Metschnikowia* sp. in Serinales. This pattern suggests several independent gene loss events of this, likely ancestral, sequence. (Figure S10).

The *CaCBP1* gene (CR_09270C_A, not to be confused with the unrelated *CBP1* of *S. cerevisiae*) is also found in most (1017/1,154) of the analyzed strains. It is, however, interesting, as it does not have a true ortholog in *S. cerevisiae*—it is homologous, but not orthologous, to another gene—*FMS1*. Loss of *CaCBP1* sequence occurred in 79 of the analyzed members of Saccharomycetales, both non- and post-WGD, but cannot be confined to any single clade and is likely a result of several independent gene loss events. It was also lost in some Dipodascales, including a clade containing 13 *Yarrowia* sp. (Figure S10).

## Discussion

The only two yeast species for which extensive experimental data on the Rtg pathway are currently available—*C. albicans* and *S. cerevisiae*—are separated by a considerable evolutionary distance of at least 234 MYA according to the time tree calculated using phylogenomic data (Opulente et al. 2024), and show significant differences in their physiology, including energy metabolism and mitochondrial biology. *S. cerevisiae* belongs to yeasts exhibiting the Crabtree effect—preference for fermentative energy production in high glucose concentration even in the presence of oxygen (Hagman et al. 2013; Pfeiffer and Morley 2014; Williams et al. 2015; Nespolo et al. 2020). In Crabtree-positive yeasts, the TCA cycle is the main source of α-ketoglutarate, and glycolysis serves as the source of ATP. Consequently, *S. cerevisiae* is a *petite*-positive yeast that can survive without functional mtDNA and oxidative phosphorylation. *Candida albicans*, on the other hand, is a *petite*-negative and Crabtree-negative species that prefers respiration (Askew et al. 2009), although respiratory-deficient mutants remain viable as long as they retain the ability to maintain intact mtDNA (Łabędzka-Dmoch et al. 2021, 2022, Wenda et al. 2024).

Whereas in *S. cerevisiae* the Rtg transcription factors were known as the model and best understood example of the retrograde mitonuclear signaling (Butow and Avadhani 2004; Liu and Butow 2006), earlier studies in *C. albicans* hinted at significant differences in the functions performed by this pathway. Together with several other transcription factors, such as Hms1 and Tye7, they were shown to be a key part of a regulatory circuit that controls diverse metabolic processes related to multiple aspects of commensalism and pathogenicity (Pérez et al. 2013), as well as the sphingolipid homeostasis (Moreno-Velásquez et al. 2020). In a classic example of evolutionary rewiring of transcriptional control, they partially replace Gal4p of *S. cerevisiae* as the regulators of galactose catabolism genes in cells with inhibited respiration (Dalal et al. 2016; Sun et al. 2023). These studies were, however, performed in strains with functional mitochondria and did not address their role in mitonuclear signaling. The results of a recent study (Liu et al. 2023), independently confirmed in our analysis, implicate the Rtg transcription factors in the activation of the inducible alternative oxidase (CaAox2) in response to mitochondrial respiratory dysfunction.

Our results indicate that in *C. albicans* the Rtg pathway is involved in the retrograde response to mitochondrial oxidative phosphorylation dysfunction by upregulation of transcription of relevant nuclear genes, similar to its well-known *S. cerevisiae* counterpart. Remarkably, however, there is virtually no overlap in the repertoires of the target genes between these two species. Even though we found homologs of the genes known to be activated by the retrograde response in *S. cerevisiae*, such as *CIT1, ACO1, IDH1/2* (encoding metabolic enzymes of the TCA cycle), *DLD3* (encoding 2-hydroxyglutarate transhydrogenase), or *PYC1* (encoding pyruvate carboxylase) in the majority of budding yeasts including *C. albicans* (albeit with a complex history of paralog duplication and loss), none of them were found to be activated by the CaRtg1/CaRtg3 transcription factor dimer. Similarly, the targets we found to be regulated by the *C. albicans* Rtg pathway in response to respiratory dysfunction are genes that are for the most part universally conserved in the Saccharomycotina subphylum, with a notable exception of CR_09270C_A (*CaCBP1*), lost in multiple independent events in different clades, including *S. cerevisiae* and related species. Yet, only two of them—*DIC1* and *PEX11* were found to be upregulated in respiratory-deficient strains of *S. cerevisiae*, albeit in a manner that does not depend on the Rtg transcription factors (Epstein et al. 2001; Guaragnella et al. 2018). This shows that these distant and metabolically dissimilar yeasts react to mitochondrial respiratory dysfunction in a largely different manner, albeit through a partially conserved regulatory system.

Among the genes activated by the retrograde response in *C. albicans*, of particular interest are those that encode proteins involved in the response to reactive oxygen species (oxidative) stress. An earlier study (Torelli et al. 2015) proposed a hormetic induction of antioxidant defense mediated by the Rtg pathway in *S. cerevisiae*, that was subsequently shown to involve the cooperation between the HOG and Rtg pathways (Guaragnella et al. 2019). In *C. albicans*, both, the alternative oxidase (CaAox2) and thioredoxin (CaTrx1), are directly involved in the response to oxidative stress (Helmerhorst et al. 2002; Alonso-Monge et al. 2009; Yan et al. 2009; da Silva Dantas et al. 2010; Liu et al. 2023; Wenda et al. 2024). The alternative oxidase has a complex evolutionary history. It is likely to be an ancient eukaryotic protein with origins that could be traced to the mitochondrial ancestor, but it was lost in several clades, including insects and vertebrates (Jacobs and Ballard 2022). The evolution of the alternative oxidase in the budding yeast subphylum shows a fascinating dynamic. It was likely present in the last common ancestor of Saccharomycotina as a single copy gene and subsequently underwent multiple independent episodes of gene loss (including in the last common ancestor of orders Saccharomycetales and Saccharomycodales) and duplication. Our analysis confirms a previously proposed (Schikora-Tamarit et al. 2021) strong correlation between the presence of genes encoding Complex I subunits in mtDNA and the alternative oxidase; and in *C. albicans* dysfunctional Complex I is known to generate oxidative stress (Li et al. 2011). A crucial role in mitigating oxidative stress, as well as other cellular stress conditions, was also postulated for AOX in Metazoa (Jacobs and Ballard 2022).

Upregulation of *NDE1*, encoding an external NADH dehydrogenase that oxidizes cytosolic NADH in mitochondria (Luttik et al. 1998), *DLD2* encoding a D-2-hydroxyglutarate dehydrogenase, and minor D-lactate dehydrogenase (Chelstowska et al. 1999), and *DIC1* that codes for a mitochondrial dicarboxylic acid transporter (Epstein et al. 2001; Punzi et al. 2018), *MLS1* encoding a peroxisomal malate synthase that participates in the glyoxylate cycle (Hartig et al. 1992; Kunze et al. 2002), and *PEX11* encoding another peroxisomal protein—a peroxin involved in peroxisome biogenesis and medium-chain fatty acid oxidation (Marshall et al. 1996; van Roermund et al. 2000; Gurvitz et al. 2001) demonstrates how the Rtg pathway reshapes cellular energy/redox metabolism in response to mitochondrial dysfunction. This aspect of the response shows some similarities between *C. albicans* and *S. cerevisiae*, as *DIC1* and *PEX11* are upregulated in the respiratory-deficient ρ^0^ strains in the latter, albeit by a different mechanism that does not involve the Rtg1/Rtg3 transcription factors (Epstein et al. 2001). In *S. cerevisiae*, another Rtg-independent mechanism, involving the Gcn4 transcription factor and Ssy1-Ptr3-Sys5 amino acid sensor, was shown to control the outward transporter Ato3 in ρ^0^ cells to restore balance between α-ketoglutarate and ammonium in respiratory deficient cells by removing excess ammonium (Guaragnella and Butow 2003).

A novel and very interesting facet of the mitonuclear retrograde response in *C. albicans* revealed in our results is related to the process of mitophagy and interactions between mitochondria and the cytoplasmic membrane system. *MDM34* encodes a protein of the mitochondrial outer membrane that is a part of the ERMES complex that tethers mitochondria to the ER and is also involved in their contact with peroxisomes (Kornmann et al. 2009; Cohen et al. 2014). Interactions between mitochondria and peroxisomes were revealed in the studies of HOG-dependent response to the hyperosmotic stress in *S. cerevisiae* that also involves the Rtg pathway (Guaragnella et al. 2021; Di Noia et al. 2023). Membrane interactions mediated by the ERMES complex are involved in mitophagy—a selective autophagy mechanism providing quality control by removing damaged mitochondria (Böckler and Westermann 2014). Involvement in mitophagy was also demonstrated in *S. cerevisiae* for the mitochondrial type 2C protein phosphatase encoded by *PTC6* (*AUP1*) (Tal et al. 2007; Journo et al. 2009), which is also a retrograde target in *C. albicans*. Studies in *S. cerevisiae* also demonstrated that mitophagy is reduced in mutants deficient in Rtg signaling (Torelli et al. 2015), providing further support for the connection between these mechanisms.

Earlier work performed in respiratory competent *C. albicans* indicated that the Rtg pathway is involved in the processes related to infectivity, filamentous growth and niche adaptation, crucial for the commensal/opportunistic pathogen lifestyle of this yeast species (Pérez et al. 2013; Yan et al. 2014). The link between mitochondrial respiration and pathogenicity in *C. albicans* is well established, and the capability of adapting cellular metabolism to different stress conditions is an important aspect of this connection (Mayer et al. 2013; Sun et al. 2019; Liu et al. 2023). The involvement of mitochondria in cell wall remodeling, Fe-S cluster homeostasis, iron sensing, and endomembrane trafficking is also implicated in the infectivity of other, evolutionary more distant fungi, such as *Aspergillus fumigatus* and *Cryptococcus neoformans* (Black et al. 2021). Modulation of stress response, in particular to the osmotic stress, depending on respiratory capacity is observed in *S. cerevisiae* (Di Noia et al. 2023, 2024) as well as in *C. albicans* (Alonso-Monge et al. 2009). The role of the Rtg pathway in these processes and its involvement in mitonuclear retrograde signaling can thus be viewed as different aspects of the same broad function, two sides of the same coin.

Altogether, these results indicate that despite significant changes in the repertoire of target genes, and the composition of the retrograde pathway (loss of regulatory factors Rtg2 and Mks1 during the evolution of Serinales), its broad function in the transcriptional response to mitochondrial dysfunction and the oxidative stress is conserved between *S. cerevisiae* and *C. albicans*. Specific adaptations reflecting broad differences in the physiology of these distant yeast species are reflected mainly in different repertoires of the target genes regulated by the Rtg transcription factors. Such an evolutionary scenario is consistent with a model proposing strong selection of the conservation of the overall function of a transcription control pathway with a weaker selection on the specific genes it targets that was previously proposed in Ascomycota (Habib et al. 2012). Our analysis shows that the genes that are controlled by the Rtg pathway in *S. cerevisiae*, *C. albicans,* or both, underwent many episodes of gene duplication and loss during the evolution of budding yeasts.

In contrast to the changing landscape of the target genes, the main components of the Rtg pathway in budding yeasts show stronger conservation. The origin of the pathway itself was likely the single gene duplication that resulted in the two paralogous transcription factors Rtg1 and Rtg3. This event occurred during the evolution of the subphylum Saccharomycotina, after the split of the three earliest branching orders—Lipomycetales, Trigonopsidales, and Dipodascales that still contain a single Rtg ortholog—and the ancestor of the remaining lineages, about 234 MYA according to the time tree estimation based on recent phylogenomic data (Opulente et al. 2024). As orthologs of Rtg3, but not of Rtg1, can be found in the genomes of other Fungi, and even in other taxa, such as Metazoa, the ancestral Rtg factor was likely closer to Rtg3. This is consistent with the observation that Rtg3, but not Rtg1, has a functional independent transcription activation domain (Rothermel et al. 1997), and with our results demonstrating that in *C. albicans* nuclear localization of Rtg1 depends on Rtg3, but not vice versa.

In the absence of experimental data from early branching yeasts, it is not clear whether the ancestral single Rtg protein is involved in the retrograde response to mitochondrial dysfunction. Paralogous duplication of genes encoding regulatory proteins is usually rapidly followed by functional reassignment (Thompson et al. 2013). A sequence fulfilling orthology criteria to yeast Rtg3 can be identified in diverse eukaryotic lineages. The human homolog of Rtg3p is the microphthalmia-associated transcription factor (MITF) (Goding and Arnheiter 2019), which is not involved in mitonuclear signaling that in Metazoa evolved independently and uses different mechanisms (Butow and Avadhani 2004; Jazwinski 2014; English et al. 2020).

The only group of yeasts that clearly lost the Rtg transcription factors is a single clade consisting of all *Starmerella* and four of the *Wickerhamiella* species. These yeasts have unique physiology related to their ecology—they occupy sugar-rich environments associated with flowers and pollinating insects, where sucrose is the predominant carbon source. They underwent dynamic evolution with extensive gene gains (including HGT events) and losses, including lost capability of alcoholic fermentation (Vandeputte et al. 2025). The accelerated gene loss in this clade was recently associated with a mutator phenotype related to impaired DNA repair (Gonçalves et al. 2025). *Starmerella* is considered more as a resource specialist organised in microbial consortia, capable of utilising a broad spectrum of carbohydrates, easily switching between respiration and fermentation (reacquired in most species) depending on sugar and oxygen access whereas *Wickerhammiella* mostly lost fermentation ability and specialized in the utilization of a narrow spectrum of carbohydrates, relying on intracellular sucrose metabolism (Gonçalves et al. 2022; Vandeputte et al. 2025). It is thus tempting to postulate a connection between these unusual physiological and ecological traits and the loss of Rtg orthologs, but in the absence of any experimental data concerning mitonuclear signaling in these yeasts any such link would be purely speculative.

Another major event in the evolution of yeast retrograde signaling was the loss of two important regulatory proteins—the activator Rtg2p and the negative regulator Mks1p. In *S. cerevisiae* the two proteins provide the switch that regulates the Rtg pathway by interacting with each other. Rtg2p works by binding the negative regulator Mks1p, which in turn keeps it from interacting with Rtg1p and Rtg3p (Liu et al. 2003). The interaction of Rtg2p and Mks1p is promoted by low ATP levels. Rtg2 is well conserved and appears to be ancestral in budding yeasts and is also present in some other lineages of Ascomycota, confirming earlier observations (Liu and Butow 2006). The sequence of Mks1 is less conserved, but its orthologs can be identified in at least some members of early branching orders of Saccharomycotina, also suggesting that it is ancestral in this clade. Genes encoding both these proteins were lost once, probably simultaneously during the evolution of the order Serinales in the ancestor of a majority of its members, including *C. albicans*. Further experimental work will be necessary to identify the mechanism regulating the Rtg transcription factors in this group. Interestingly our results, as well as the earlier study of Liu et al. (2023), show that unlike in *S. cerevisiae*, in *C. albicans*, the Rtg1 and Rtg3 transcription factors do not change their subcellular localization upon the activation of the pathway and remain in the nucleus all the time. One might speculate that this difference in the mode of activation of the retrograde transcription factors and the absence of the Rtg2 and Mks1 regulatory factors might be linked, but in the absence of more extensive experimental studies this must be considered as a conjecture.

Overall, our analysis provides a scenario for the origin and evolution of the retrograde mitonuclear signaling in budding yeasts. *In silico* analysis profiting from the wealth of genome sequences indicates how paralogous duplications and gene loss events shaped this regulatory system. Our experimental results link it to a plethora of metabolic responses to stress, including reactive oxygen species mitigation and mitophagy. One is tempted to speculate that the Rtg pathway originated in yeasts as a universal regulatory system responding to a variety of different stress and metabolic conditions. The Hog1 MAP kinase integrating osmolarity homeostasis with respiratory metabolism and retrograde signaling is an example of such conserved mechanism that is present both in *S. cerevisiae* (Guaragnella et al. 2013, 2021, Di Noia et al. 2023, 2024) and *C. albicans* (Alonso-Monge et al. 2009). Those aspects of this response that were described in *S. cerevisiae* but are not present in *C. albicans,* could be related to the highly specialized fermentative metabolism of the former, whereas the importance of oxidative stress response through the alternative oxidase and other factors in the latter reflect the specific adaptations of this mostly respiratory commensal and opportunistic pathogen. Finally, as the presence of an orthologous gene does not constitute a proof of conserved function, many questions concerning the evolution of this system require experimental work expanding beyond the few model species that had been studied so far.

## Materials and methods

### *C. albicans* culture and strain construction

Genetic manipulations were performed in the *C. albicans* BWP17 strain (Wilson et al. 1999). The strains used in this study are listed in Table S1. The yeast strains were grown in the YPD or YPGal medium supplemented with uridine (80 μg/ml) at 30 °C.

Knockout strains were obtained by the PCR-based gene targeting strategy (Walther and Wendland 2008) using deletion cassettes carrying the *CaHIS1* and synthetic *SAT1* gene as described in Wenda et al. (2024). Double deletion strains were obtained by four rounds of homologous recombination with the use of *CaHIS1*, *CaURA3*, *CaARG4*, and *CaSAT1* genes as selection markers. Strains with double deletion expressing GFP fusion proteins were obtained by FOA counterselection (1 mg/ml FOA) and following integration of the *GFP-CaURA3* gene in desired locus (*CaRTG1* or *CaRTG3*) with the aid of CRISPR editing as described before (Vyas et al. 2015).

Strains expressing the CaRtg1-HA fusion were obtained by homologous integration of the PCR amplified (all primers used for PCR amplification are listed in Table S2) fragment containing an HA tag and *CaURA3* gene as a selection marker in the appropriate genetic background (*ΔCappr13*, *ΔCaaep3* or WT with one *CaRTG1* allele removed, by exchange for *CaARG4* gene). The resulting strains express the CaRtg1-HA as the only copy present in the genome. Analogous manipulations were carried out for CaRtg3-HA construction.

### Fluorescence microscopy and localization studies

GFP expressing strains were constructed as described before (Wenda et al. 2024). Briefly, the fragment coding for GFP with the selection marker was amplified by PCR and integrated into the genome of wild type and respiratory-deficient strains. The *CaRTG3_Psites_* allele with six serines S_231_, S_235_, S_247_, S_251_, S_398_, S_401_ exchanged into alanines was obtained by three rounds of site-directed mutagenesis of plasmid *pFA-RTG1-CaURA3* followed by PCR amplification of the *CaRTG3_Psites_-CaURA3* on the plasmid template and homologous integration into the genome.

Templates for MBP-CaRtg1 and MBP-CaRtg3 protein synthesis were obtained by PCR amplification of *CaRTG1* and *CaRTG3* sequences flanked by SalI and NotI sites, followed by cloning into *pETMM41-MBP* vector (Addgene). Subsequently both vectors were subjected to two (*pETMM41-MBP-Cartg1*) or four (*pETMM41-MBP-Cartg3*) rounds of site-directed mutagenesis to exchange CTG codons into TCG, important for in vitro protein synthesis.

The CaRtg1-mCherry expressing strains were obtained as follows. Fragment including codon optimized mCherry was subcloned from the *pAYCU257* (Defosse et al. 2018) into the *pFA-CaARG4* vector. In the second step fragment containing mCherry with *TRP1* terminator and *CaARG4* marker was amplified by PCR with flanking fragments homologous to 3′ coding sequence and the terminator of the *CaRTG1*. The yeast cells transformed with such fragment, selected for the *CaARG4* allele were screened by diagnostic PCR and microscopy observations. Yeast cells expressing GFP or mCherry fusions were grown to OD_600_ 1.2–1.5. Hoechst 33342 trihydrochloride hydrate (Life Technologies) staining was performed in 20 μg/ml PBS-based solution for 5 min at room temperature. Stained cells were observed using the OLYMPUS IX 81 fluorescent microscope with UPlanSApo O 60 × and 100 × lenses and a filter set (GFP ex = 485 nm, em 525 nm; mCherry ex = 562 nm, em = 641 nm; Hoechst ex = 387 nm, em = 440 nm). Images were taken with an EMCCD ORCA R2 camera (Hamamatsu) and analyzed using the FIJI software (Schindelin et al. 2012).

### GFP reporter assay

For the reporter assay, GFP upstream of *CaURA3* was amplified on the template of *pFA-GFP-CaURA3* vector (Walther and Wendland 2008) integrated downstream of the *CaAOX2* promoter in WT, *ΔCartg1, ΔCartg3, ΔCaaep3, ΔCaaep3 ΔCartg1,* and *ΔCaaep3 ΔCartg3* strains. Clones were selected on plates lacking uracil and verified by diagnostic PCR.

### Immunoblotting

Immunoblotting was performed as described before (Wenda et al. 2024). Detection was carried out with mouse anti-HA primary antibodies (Invitrogen CAT #26183) and goat anti mouse HRP secondary antibodies (Calbiochem). Western blot analysis was performed with FIJI software (Schindelin et al. 2012). CaRtg1-HA and CaRtg3-HA levels were relatively quantified using normalization to total protein content determined by Ponceau S staining. Statistical analysis of relative protein levels (ANOVA and students *t*-test) was performed using RStudio and R (version 4.3.1), and the plots were generated using the *ggplot2* package (Wickham 2016).

### Isolation of RNA and northern blot

Isolation of *C. albicans* RNA and northern blotting were performed as described previously (Wenda et al. 2024).

### Chromatin immunoprecipitation and sequencing (ChIP-seq)

ChIP-seq experiments were performed according to the published protocol (Znaidi et al. 2014) with some modifications. Cells were grown in a YPGal medium until an OD_600_ = 1 and fixed by adding 2.8 ml 37% formaldehyde per 100 ml of culture and incubated for 20 min at room temperature with agitation. Crosslinking was stopped by adding 5 ml 2.5 M glycine for 2 min, and cells were harvested by centrifugation for 5 min at 2,465 *g*. Cell pellets were washed three times with 20 ml TBS buffer (20 mM Tris-HCl, 150 mM NaCl), centrifuged for 5 min at 2,465 *g* and frozen in liquid nitrogen.

For the chromatin protein complexes extraction, the cell pellet thawed on ice was resuspended in 1.4 ml of lysis buffer (50 mM HEPES KOH, 140 mM NaCl, 1 mM EDTA, 1% TRITON X-100, 0.1% NA-deoxycholate) supplemented with protease inhibitors and 1 mM PMSF. The cell extracts were prepared by bead beating in FastPrep-24 (MP Biomedicals) (6 runs with 6 m/s speed, 1 min each and 5 min intervals in between). Pellets were separated from supernatants by 10 min 7,500 *g* centrifugation. Supernatants containing soluble protein were discarded and the chromatin-containing pellet was resuspended in 1.4 ml of fresh buffer and sonicated (Bioruptor Diagenode sonicator). The shearing was controlled by loading the chromatin samples on 2% agarose gel in between to obtain an optimal size of ≤500 bp.

The soluble chromatin was separated by centrifugation, 50 μl was collected as an input sample, and 1 ml was loaded to the 50 μl magnetic beads coupled to anti-HA antibody (Thermo Scientific CAT# 88838), prewashed with lysis buffer and blocked with 0.5% BSA. Precipitation was performed overnight at 4 °C with the agitation. After the incubation, the beads were captured using a magnetic rack and washed twice with 1 ml of lysis buffer, twice with lysis buffer with high salt (50 mM HEPES KOH, 500 mM NaCl, 1 mM EDTA, 1% TRITON X-100, 0.1% NA-deoxycholate), twice with wash buffer (10 mM Tris-HCl pH 8.0, 250 mM LiCl, 0.5% NP40, 0.5% Na-deoxycholate, 1 mM EDTA) and once with TE buffer. Decrosslinking was performed overnight at 65 °C in TE buffer with SDS (10 mM Tris pH 8.0, 1 mM EDTA, 1% SDS) followed by RNAse A (Thermo Scientific) treatment at 37 °C for 2 h and Proteinase K (Thermo Scientific) digestion at 55 °C for 2 h. The DNA was extracted by phenol:chloroform:isoamyl (Roth) and isopropanol precipitated, dried, and dissolved in water. ChIP-seq analysis was performed in the Centre of New Technologies Next-Generation Sequencing Core Facility (University of Warsaw, Nova Seq 6000 (Illumina)) in paired-end mode.

### Preprocessing, batch correction, and quantification of ChIP-seq data

Reads were mapped to the haploid version of SC5314 reference genome with bowtie2 (Langmead and Salzberg 2012) using default settings. Duplicates were removed with Picard (https://broadinstitute.github.io/picard/). Peaks were identified with macs2 (Feng et al. 2012) in paired-end mode and annotated with ChIPseeker (Yu et al. 2015).

Consensus peak set was obtained by merging macs2 peaks from all samples. Peaks present in mock experiments were then subtracted with bedtools (Quinlan and Hall 2010), and the peak set was converted from BED to SAF format. The SAF file was then used to quantify reads corresponding to each peak with featureCounts (Liao et al. 2014). The read counts were then used as input for batch correction with ComBat (Johnson et al. 2007). Primary component analysis was performed in R with prcomp (R Core Team), both before and after batch correction, to assess its effectiveness (Figure S12).

Preprocessed and annotated peaks from each sample were analyzed both prior to and following batch correction. For data that did not undergo batch correction, peaks from corresponding mock experiments were first subtracted with bedtools. Subsequently, peaks from the WT strain were subtracted from mutant strains from corresponding batches, in order to obtain mutant-specific enrichments.

For batch corrected data, peaks from mock experiments were similarly subtracted. Peaks that were differentially enriched in deletion strains (relative to the WT strain) were detected via linear modeling and empirical Bayes smoothing in limma package (Ritchie et al. 2015), with *P* value < 0.05.

### Pathway enrichment analysis

Enrichment analysis was performed with GSEABase (Subramanian et al. 2005), with SlimGO pathway annotation, a *P*-value cutoff of 0.3 and q-value cutoff of 0.4. *Candida albicans* genes corresponding to all detected ChIP peaks were used as background. Enrichment results were visualized in R with enrichplot (Yu G (2025). *enrichplot: Visualization of Functional Enrichment Result*. doi:10.18129/B9.bioc.enrichplot, R package version 1.28.2, https://bioconductor.org/packages/enrichplot). For deletion strains, genes corresponding to differentially enriched peaks in batch corrected data were used as input. For the WT strain, genes corresponding to the 100 most enriched peaks (from batch 1, which had the best coverage for WT strain) prior to batch correction were used.

### Motif discovery

MEME-ChIP (Machanick and Bailey 2011) a part of the MEME Suite (Bailey et al. 2015) was used for motif detection in sequences corresponding to enriched peaks (extracted with bedtools) detected in batch-corrected data, including peaks significantly enriched in deletion strains relative to WT strain and 100 peaks with the highest average signal in WT strain. The analysis included MEME and STREME algorithms and was run for motifs with lengths of between 4 and 10 nucleotides, with *P* value cutoff of 0.05. Sequences containing canonical motifs were then subtracted from the input FASTA files, and the analysis was re-run in order to detect putative non-canonical binding motifs.

### Electrophoretic mobility shift assays

The DNA fragments of 127–209 bp were amplified by PCR on the *C. albicans* genomic DNA template, isopropanol precipitated and radiolabeled by PNK (Thermo) with [γ-^32^P]-ATP (Hartmann Analytic) as a substrate. The proteins for the binding assays were synthesized by PURExpress in vitro Protein Synthesis Kit (New England Biolabs) according to the manufacturer's instructions with *pETMM41-MBP-CaRTG1*, *pETMM41-MBP-CaRTG3* or *pETMM41-MBP* as a negative control. Two microlitres of one or both protein mixtures were used in the binding reaction. The binding reaction was performed using the 5 × EMSA binding buffer (50 mM Tris-HCl pH 8.0, 500 mM KCl, 10 mM MgCl_2_, 25% glycerol) for 20 min at room temperature. One nanogram of the labeled probe was used in a single EMSA experiment. The same amount of probe without protein or probe with MBP-tag was used as negative controls. Samples containing binding reaction and loading dye (30% glycerol, 0.25% xylene cyanol, 0.25% bromophenol blue) were separated by electrophoresis in 5% non-denaturing polyacrylamide gel containing 0.5 × TBE buffer, pH 8.35 (44.5 mM Tris-base, 44.5 mM H_3_BO_3_, 1 mM EDTA) for around 2 h at 200-220 V in a cold room. The gels were dried and exposed to the PhosphorImager screens (FujiFilm) and scanned by Typhoon FLA 9000 (GE Healthcare). Images were analyzed in FIJI software (Schindelin et al. 2012).

### Sequence databases

We used the published genome sequences of 1,154 yeast strains from 1,051 species (Opulente et al. 2024) downloaded locally and converted to NCBI format databases for use with the command line BLAST+ programs (Camacho et al. 2009). The *Saccharomyces* Genome Database (Wong et al. 2023), and *Candida* Genome Database (Skrzypek et al. 2017) were also used to retrieve and analyze sequences from the relevant species. Budding yeast mitochondrial DNA sequence analysis was performed using genome data from 353 strains (Wolters et al. 2023). Additional genome sequences of other taxa were downloaded from NCBI, and the accession numbers (assembly IDs) are provided in Table S3.

### Ortholog and paralog identification

Searching for orthologs was performed using BLAST algorithm (Altschul et al. 1990) against the amino acid (blastp) sequence database of 1,154 budding yeast strains. Identified sequences were then used to search the protein sequences of the query genome (*S. cerevisiae* or *C. albicans*), and only those that gave the original query as the top hit were retained (reciprocal best hits criterion for orthology). In the cases where no orthologs were found, this was further confirmed by searching the translated nucleotide sequences with tblastn. As the high-throughput sequencing and automatic ORF identification sometimes leads to artifacts (e.g. concatenated amino acid sequences of different proteins reported as a single entry), we performed additional checks on results failing the reciprocal best hit criterion.

For the identification of paralogs homologous to a query sequence, all the hits above the E-value threshold (adjusted depending on the conservation of the sequence of interest) were counted. Alignments and phylogenetic analyses were used to eliminate partial (two fragments of the same sequence annotated as two separate entries) and identical sequences. Python scripts used to automate the analyses, together with their results, are available at https://doi.org/10.58132/7PTVW4 as Jupyter notebooks.

### Phylogenetic analyses

The species tree, time tree, and classification into orders were taken from the analysis by Opulente et al. (2024). The tree from Wolters et al. (2023) was used for analyses involving the mitochondrial genome sequences. For new phylogenetic trees of selected proteins, likely incomplete or concatenated sequences that broke the alignments were filtered by eliminating those that were less than 0.5 × or more than 2 × the length of the query sequence. Amino acid sequences were then aligned using Clustal Omega (Sievers and Higgins 2018), and the alignments were visualized and checked in AliView (Larsson 2014) or Jalview (Waterhouse et al. 2009). For phylogenetic analysis the alignments were trimmed to remove noninformative regions in BMGE 2 (Criscuolo and Gribaldo 2010) using the BLOSUM30 similarity matrix and default parameters. Phylogenetic tree was inferred by the maximum likelihood (ML) method using IQ-TREE (Nguyen et al. 2015) with the model identified using ModelFinder (Kalyaanamoorthy et al. 2017). Branch support was assessed using the ultrafast bootstrap approximation using a hill-climbing nearest neighbor interchange (NNI) search (Hoang et al. 2018) with 1,000 repeats. The published phylogenomic tree of 1,154 yeast strains (Opulente et al. 2024) was used as the species tree, and the published mtDNA tree (Wolters et al. 2023) was used in the analyses that involved mitochondrially encoded genes. Trees were visualized using iTOL v. 6 (Letunic and Bork 2024). CLANS v. 2 (Frickey and Lupas 2004) was used for clustering of Rtg protein orthologs using the Fruchterman–Reingold graph layout algorithm in the 3D space.

## Supplementary Material

msag005_Supplementary_Data

## Data Availability

The data underlying this article are available in the University of Warsaw Research Data Repository at https://doi.org/10.58132/7PTVW4. The phylogenetic trees are available in the Interactive Tree of Life at https://itol.embl.de/shared/pgolik and can be accessed with the user ID pgolik. NGS reads are available in the European Nucleotide Archive (ENA) and can be accessed with the study accession number PRJEB93920.
