# Supplementary material for: Mitochondrial retrograde control of transcription evolves with respiratory stress, metabolic adaptation, and virulence in budding yeasts
Source: Mol Biol Evol. 2026 Jan 9;43(2):msag005. doi: 10.1093/molbev/msag005 (PMC12862490; doi:10.1093/molbev/msag005)
Supplement: msag005_Supplementary_Data [file msag005_supplementary_data.pdf]

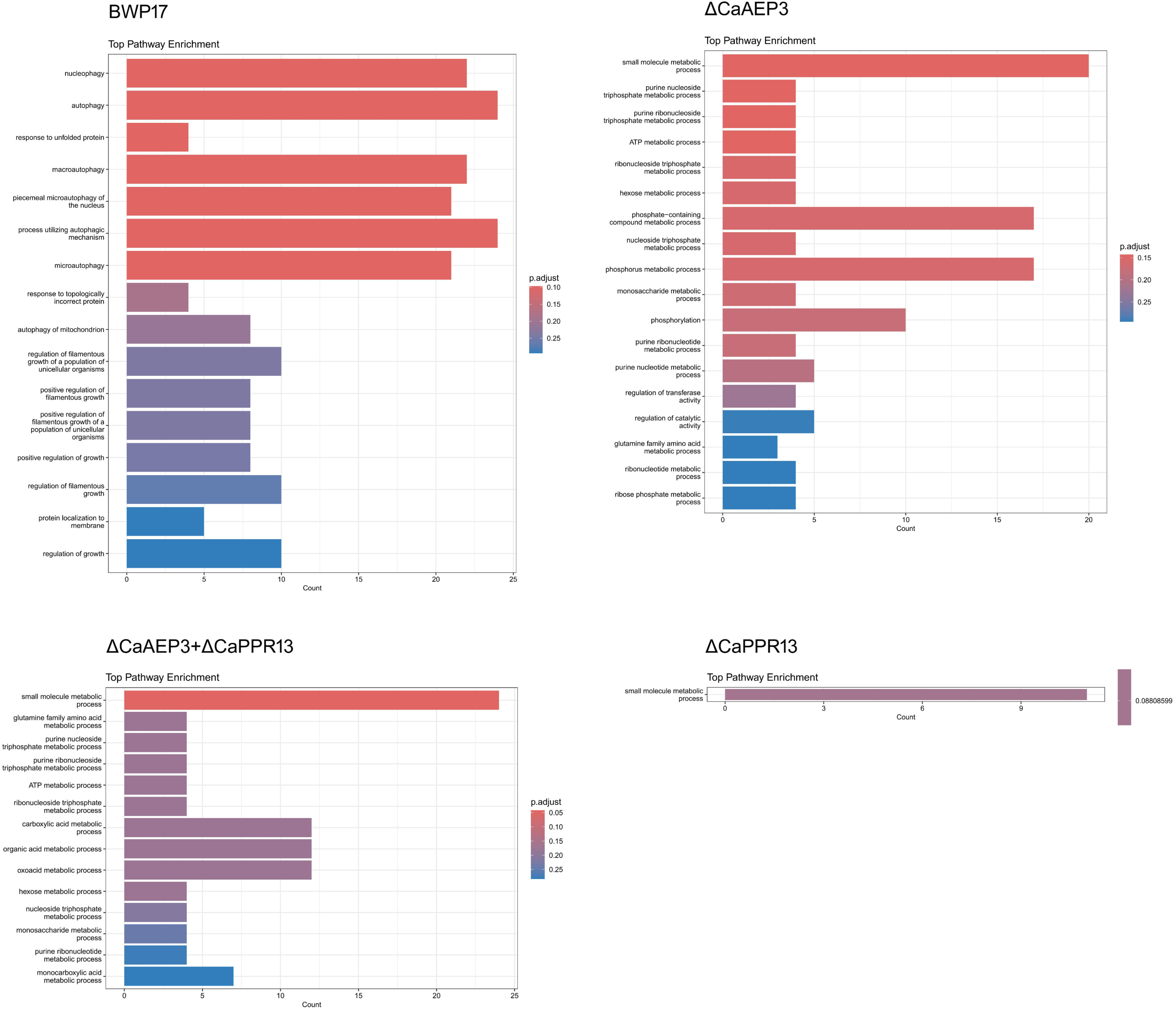

Supplementary Figure S1. Pathway enrichment results from ChIP-seq experiments for WT *C. albicans* strain (BWP17), *ΔCaAEP3* strain, *ΔCaPPR13* strain and both deletion strains combined

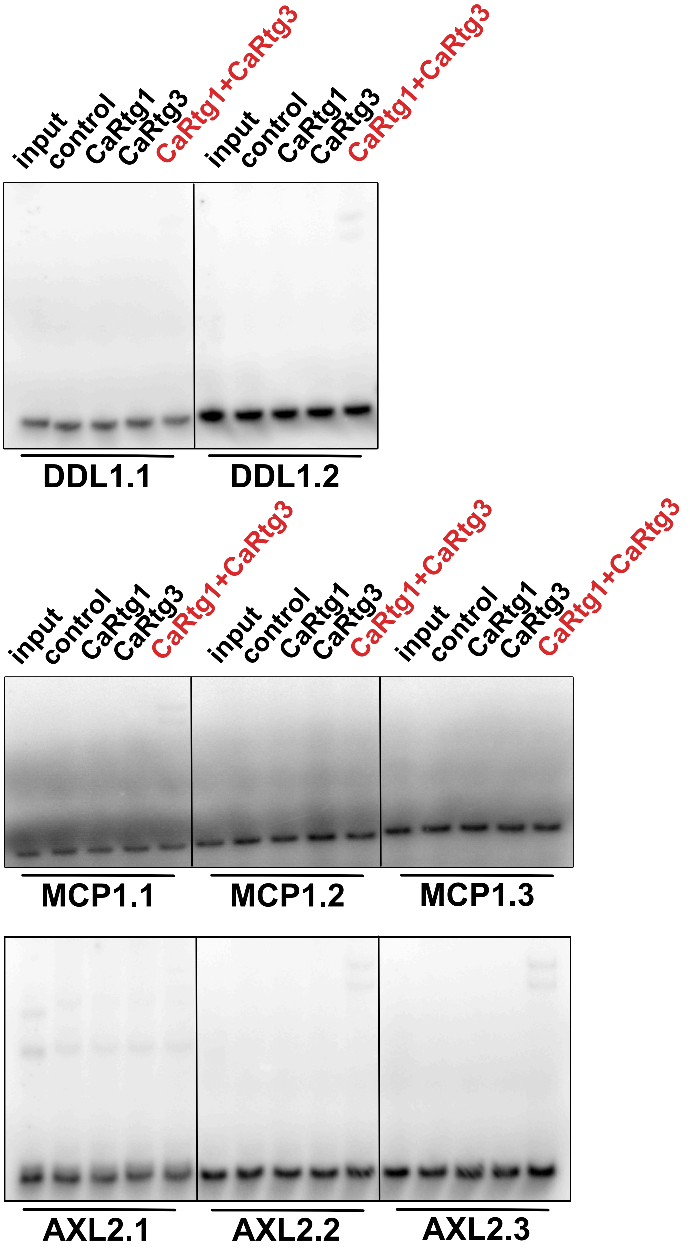

Supplementary figure S2. CaRtg1/CaRtg3 heterodimer does not bind to promoter regions of *CaDDL1*, *CaMCP1* and *CaAXL2* genes.

Binding of CaRtg1-MBP/CaRtg3-MBP (CaRtg1+CaRtg3), but not CaRtg1-MBP (CaRtg1), CaRtg3-MBP (CaRtg3) or MBP tag (control) to the GTCA containing motifs of the *CaDDL1*, *CaMCP1*, and *CaAXL2* and respective controls. *CaAXL2* promoter fragments binding to the CaRtg1/CaRtg3 heterodimer is considered unspecific, due to the stronger binding signal of the control fragment (AXL2.3), compared to the GTCA containing probes.
EMSA of the radioactively labelled DNA probes was performed in 5% non-denaturing polyacrylamide gel with CaRtg1-MBP, CaRtg3-MBP, CaRtg1-MBP+CaRtg3-MBP, MBP tag (control) or without protein (input).

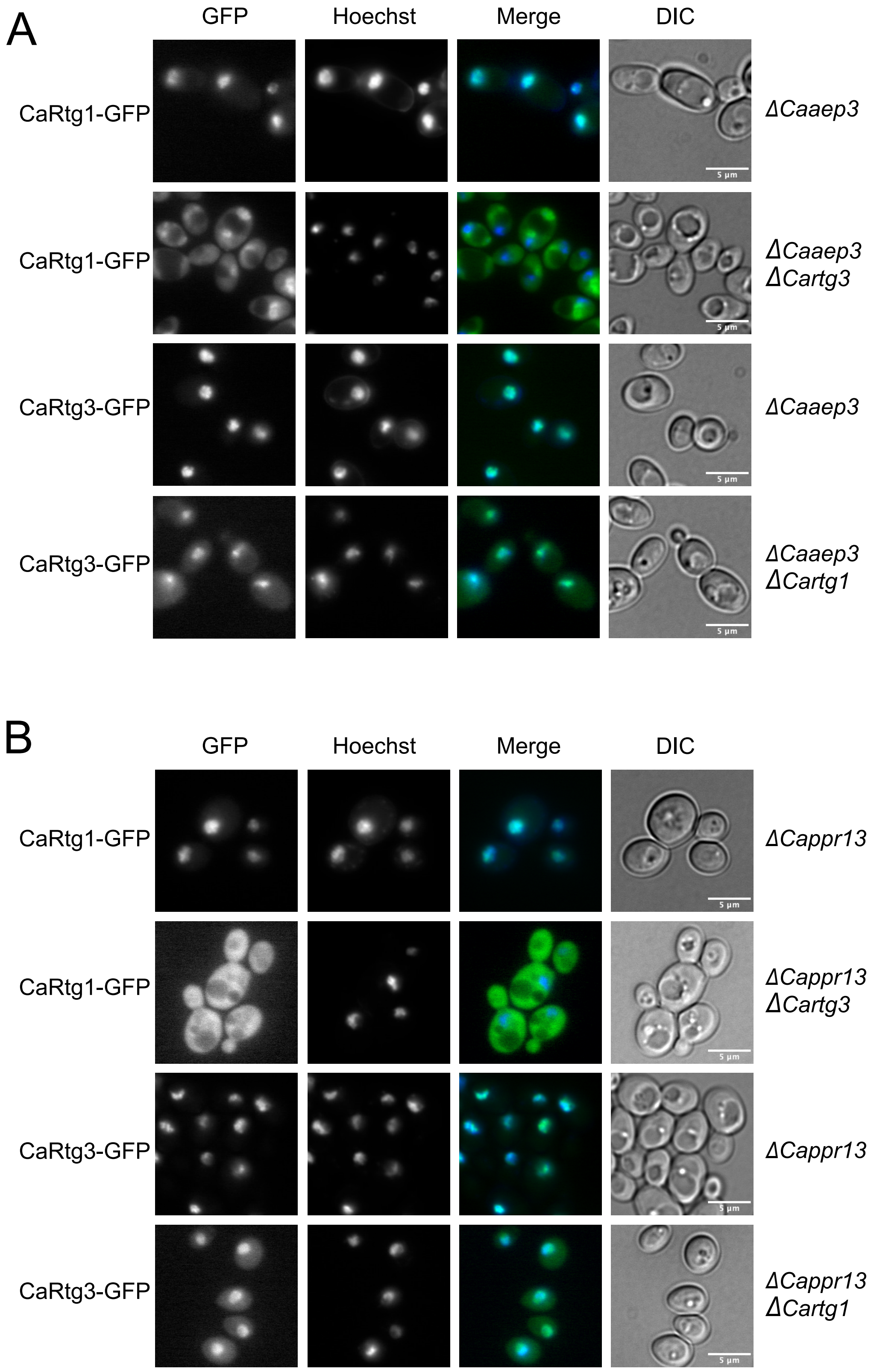

Supplementary figure S3. Respiratory competence does not influence CaRtg1-GFP and CaRtg3-GFP cellular localization.

Fluorescent imaging of CaRtg1-GFP in Δ*Caaep3,* Δ*Caaep3*Δ*Cartg3* and CaRtg3-GFP in Δ*Caaep3,* Δ*Caaep3*Δ*Cartg1* (in **A**) CaRtg1-GFP in Δ*Cappr13,* Δ*Cappr13*Δ*Cartg3* and CaRtg3-GFP in Δ*Cappr13,* Δ*Cappr13*Δ*Cartg1*Δ (in **B**).
Nuclear localization of CaRtg1-GFP and CaRtg3-GFP is shown regardless of respiratory competence of investigated strains. In the absence of CaRtg3, CaRtg1-GFP exhibits extranuclear localization.
In contrast, in Δ*Cartg1* strain CaRtg3-GFP remains in the nucleus.
Nuclei are labelled with Hoechst 33342, DIC (Differential Interference Contrast) is shown as the reference image. The scale bar corresponds to 5 µm.

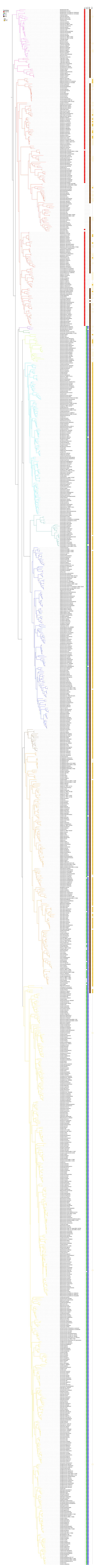

Supplementary figure S4. Orthologs of the major components of the retrograde pathway: the Rtg1 and Rtg3 transcription factors (TFs), and the regulatory factors Rtg2 and Mks1 in the phylogenomic tree of 1154 yeast strains from 1051 species (Opulente et al. 2024). Alternative presentation of the tree in Fig. 6. See the Interactive Tree of Life at https://itol.embl.de/shared/pgolik for the interactive version.

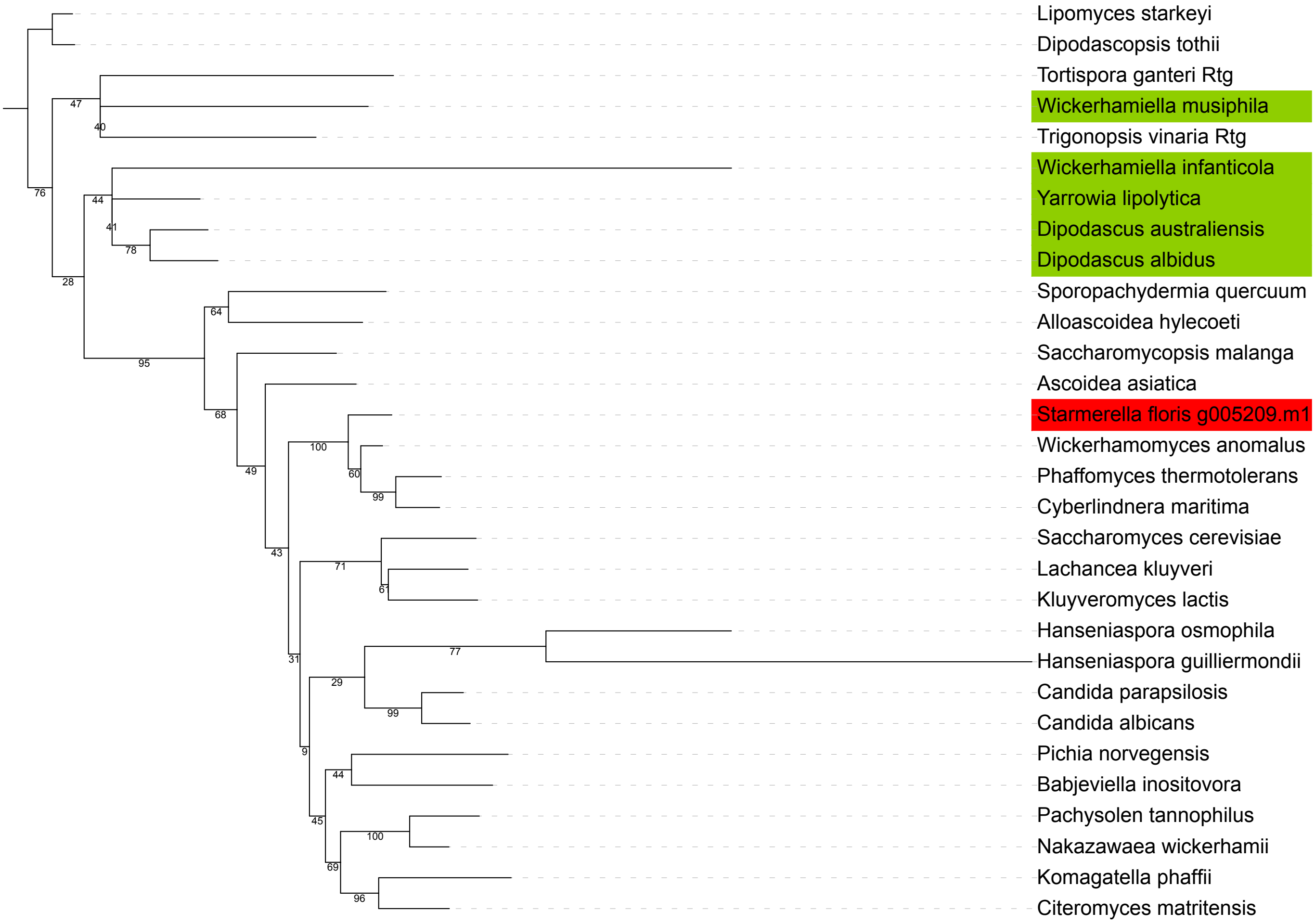

Supplementary Figure S5. The putative Rtg ortholog in *Starmerella floris* (highlighted in red) is related to Rtg3 of Phaffomycetales rather than to the Rtg proteins of other Dipodascales (highlighted in green). The sequence of g005209.m1 protein from *St. floris* that is orthologous to Rtg3 and 29 Rtg3 or single Rtg protein sequences representing all orders of Saccharomycotina were aligned, the alignment was trimmed to remove noninformative regions (leaving 183 aa remaining), and the ML tree was obtained as described in Materials and Methods. The tree was rooted using Lipomycetales as outgroup. Numbers indicate bootstrap values for nodes.

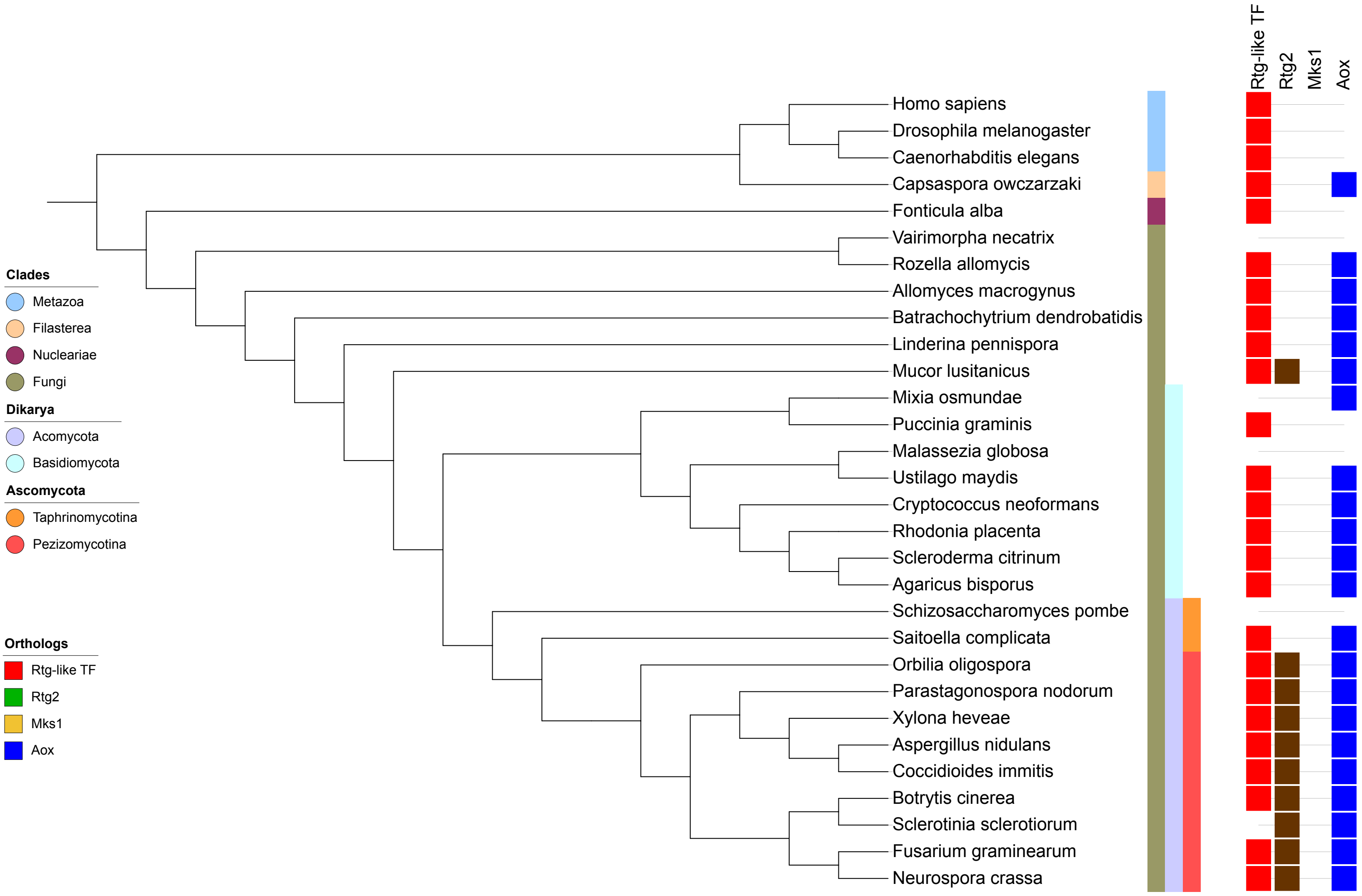

Supplementary Figure S6. Orthologs of the major components of the retrograde pathway: the Rtg1 and Rtg3 transcription factors (TFs), the regulatory factors Rtg2 and Mks1, and the alternative oxidase (Aox) in other Fungi and Opistokontha.

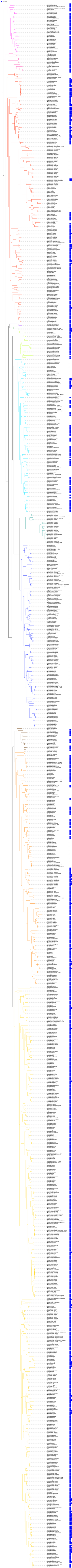

Supplementary figure S7. Orthologs of the alternative oxidase (AOX) in the phylogenomic tree of 1154 yeast strains from 1051 species (Opulente et al. 2024). The height of the bar and numbers correspond to the number of paralogous Aox sequences in a genome (0 to 4) Alternative presentation of the tree in Fig. 8A. See the Interactive Tree of Life at https://itol.embl.de/shared/pgolik for the interactive version.

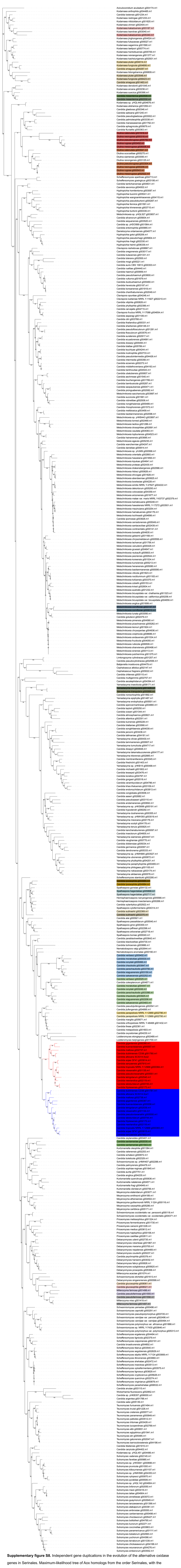

Supplementary figure S8. Independent gene duplications in the evolution of the alternative oxidase genes in Serinales. Maximum-likelihood tree of Aox homologs from the order Serinales, with the bootstrap values indicated. Alternative presentation of the tree in Fig. 9. See the Interactive Tree of Life at https://itol.embl.de/shared/pgolik for the interactive version.

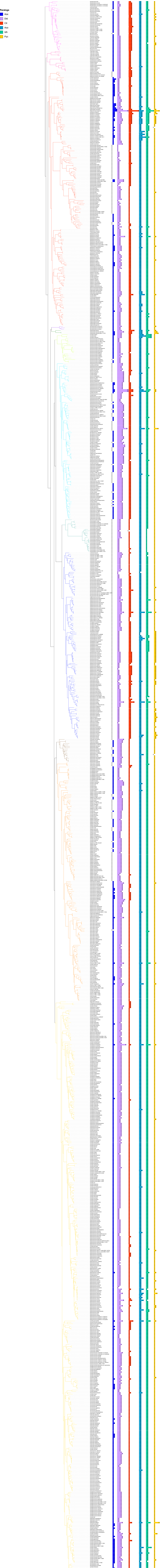

Supplementary figure S9. Orthologs of the canonical targets of the Rtg pathway (as identified in *S.* cerevisiae) and the alternative oxidase (Aox) in the phylogenomic tree of 1154 yeast strains from 1051 species (Opulente et al. 2024). The height of the bar and numbers correspond to the number of paralogs. See the Interactive Tree of Life at https://itol.embl.de/shared/pgolik for the interactive version.

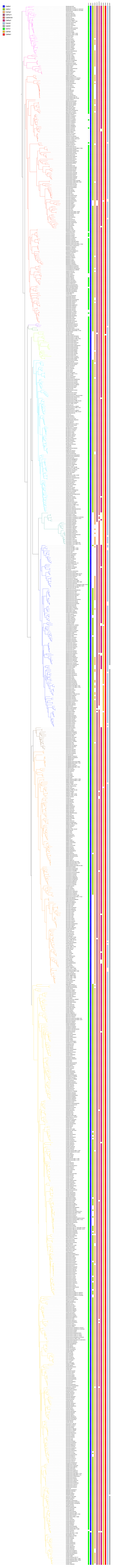

Supplementary figure S10. Orthologs of the targets of the Rtg pathway identified in *C. albicans* in this work in the phylogenomic tree of 1154 yeast strains from 1051 species (Opulente et al. 2024). See the Interactive Tree of Life at https://itol.embl.de/shared/pgolik for the interactive version.

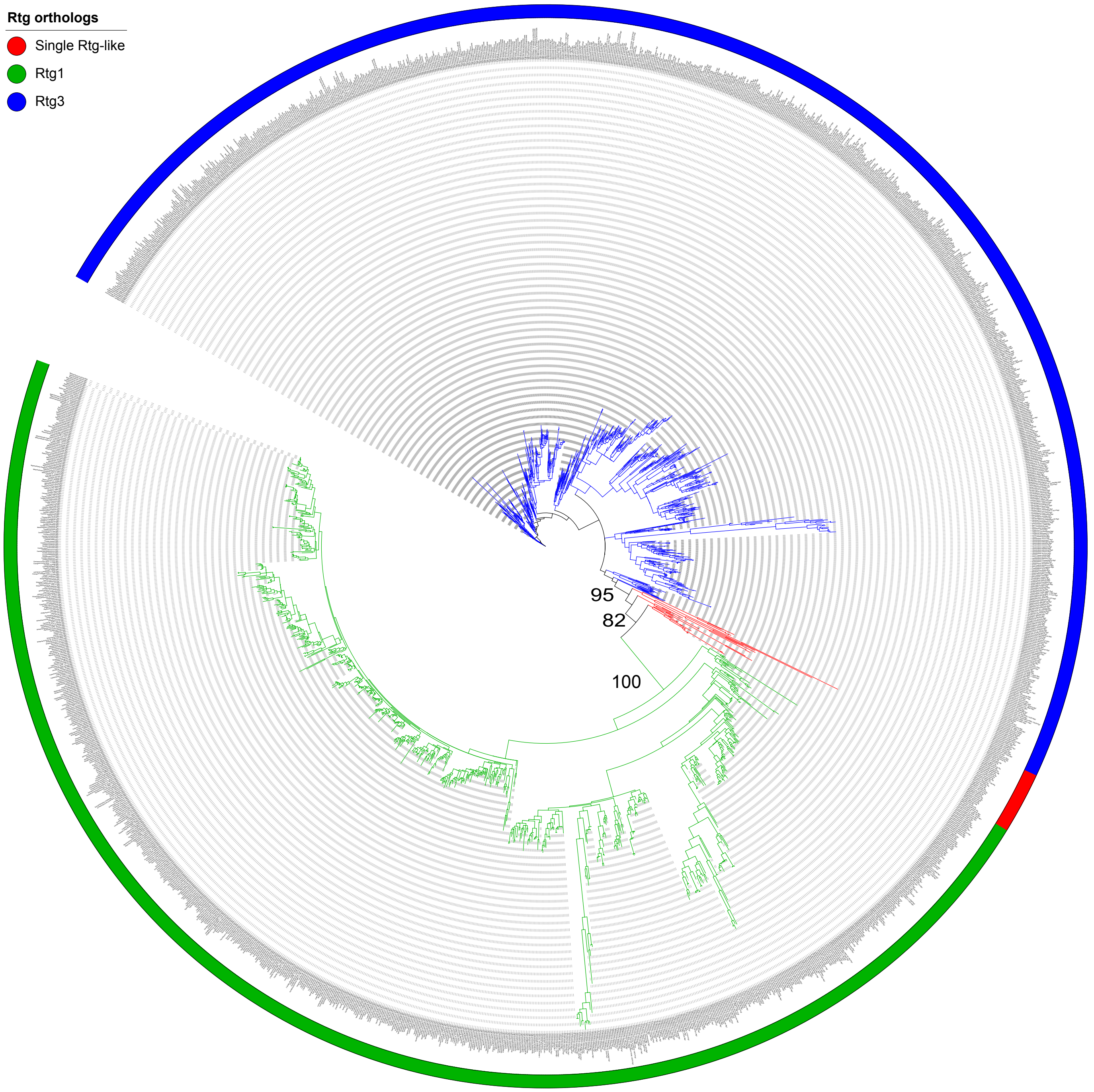

Supplementary Figure S11. Maximum-likelihood tree of the Rtg orthologs. Numbers indicate bootstrap values for selected nodes. The orthologs of either Rtg1 or Rtg3 of S. cerevisiae (2017 sequences) were filtered to remove incomplete and/or concatenated sequences, resulting in a set of 1804 sequences that were aligned. The alignment was trimmed to remove noninformative regions (leaving 115 aa remaining) and the consensus ML tree of 1000 bootstrap replicates was obtained as described in Materials and Methods. This is an **unrooted** tree, corresponding to the tree in Fig. 7 in the main text (which was rooted using Lipomycetales as outgroup).

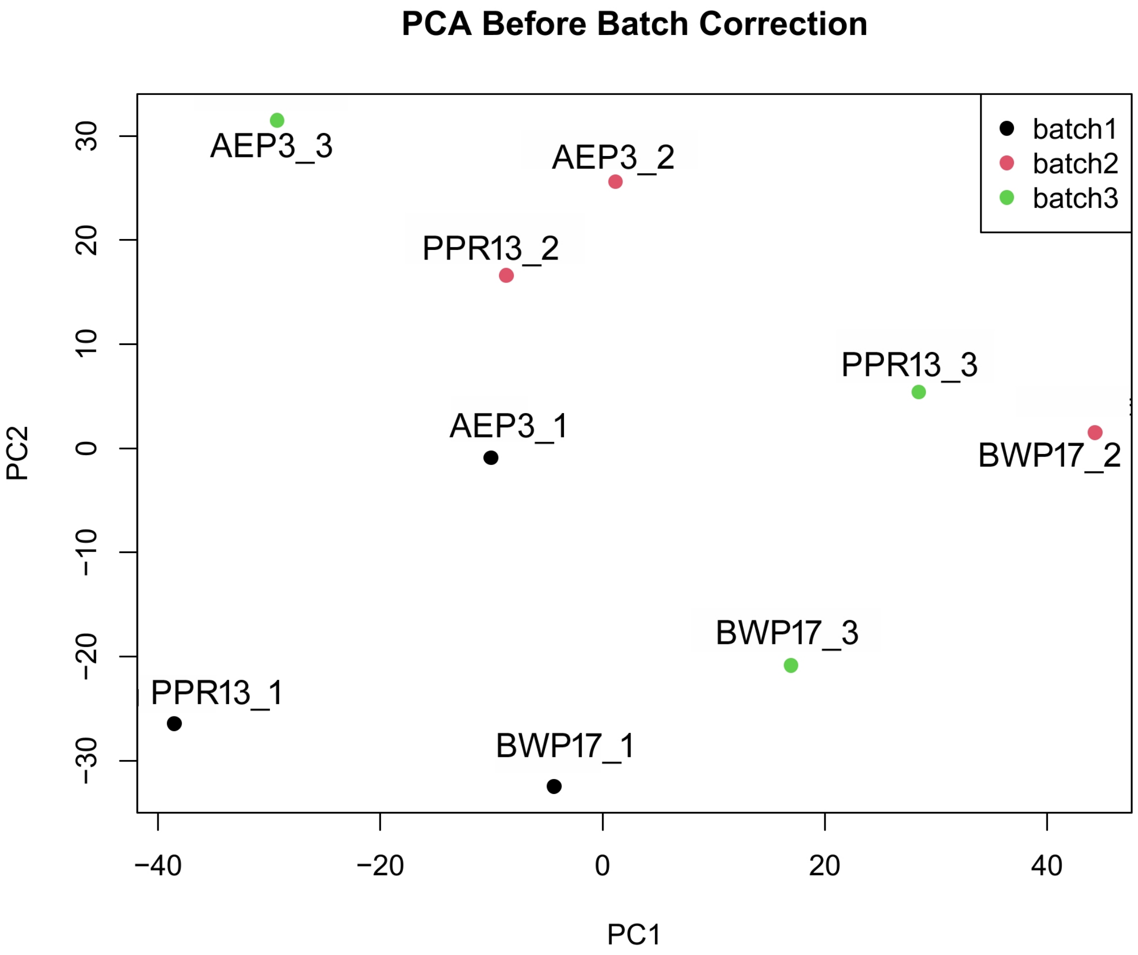

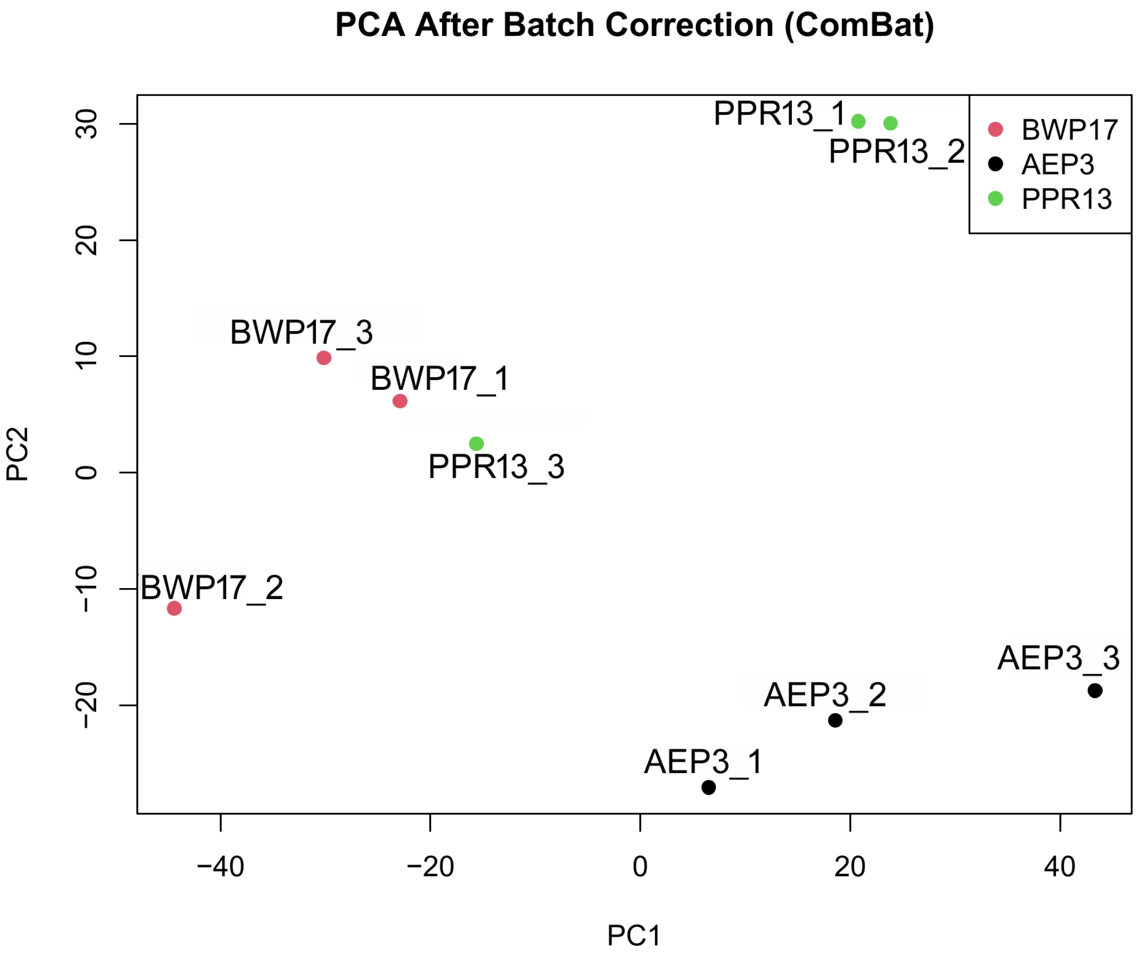

Supplementary Figure S12. PCA analysis for all three replicates of ChIP-seq experiments, before and after batch correction with ComBat

SupplementaryTable S1. Yeast strains and plasmids used in this study

Name	Genotype*	Source
BWP17	*arg4/arg4; his1/his1; ura3/ura3*	Wilson *et al.* 1999
ΔCaaep3	*AEP3::CaSAT1^{R}/AEP3::HIS1*	Wenda *et al.* 2024
ΔCappr13	*PPR13::CaSAT1^{R}/PPR13::HIS1*	Wenda *et al.* 2024
ΔCaaep3 RTG1HA	*AEP3::CaSAT1^{R}/AEP3::HIS1; RTG1::ARG4/RTG1::RTG1-HA-URA3*	This study
ΔCappr13 RTG1HA	*PPR13::CaSAT1^{R}/PPR13::HIS1*<br>*RTG1::ARG4/RTG1::RTG1-HA-URA3*	This study
CaRtg1-HA	*RTG1::SAT1^{R}/RTG1::RTG1-HA-URA3*	This study
CaRtg3-HA	*RTG1::SAT1^{R}/RTG1::RTG1-HA-URA3*	This study
ΔCaaep3 RTG3HA	*AEP3::CaSAT1^{R}/AEP3::HIS1*<br>*RTG3::ARG4/RTG3::RTG3-HA-URA3*	This study
ΔCappr13 RTG3HA	*PPR13::CaSAT1^{R}/PPR13::HIS1*<br>*RTG3::ARG4/RTG3::RTG3-HA-URA3*	This study
ΔCartg1	*RTG1::SAT1^{R}/RTG1::HIS1*	This study
ΔCartg3	*RTG3::SAT1^{R}/RTG3::HIS1*	This study
ΔCartg1 ΔCaaep3	*RTG1::ARG4/RTG1::URA3*<br>*AEP3::CaSAT1^{R}/AEP3::HIS1*	This study
ΔCartg1 ΔCappr13	*RTG1::ARG4/RTG1::URA3*<br>*PPR13::SAT1^{R}/PPR13::HIS1*	This study

ΔCartg3 ΔCaaep3	*RTG3::ARG4/RTG3::URA3*<br>*AEP3::CaSAT1^R/AEP3::HIS1*	This study
ΔCartg3 ΔCappr13	*RTG3::CaARG4/RTG3::URA3*<br>*PPR13::CaSAT1^R/PPR13::HIS1*	This study
ΔCartg1 ΔCaaep3+CaRTG1	*RTG1::ARG4/P_{RTG1}-RTG1-T_{RTG1}-URA3*<br>*AEP3::CaSAT1^R/AEP3::HIS1*	This study
ΔCartg3 ΔCaaep3+CaRTG3	*RTG3::ARG4/P_{RTG3}-RTG3-T_{RTG3}-URA3*<br>*AEP3::CaSAT1^R/AEP3::HIS1*	This study
CaRtg1-GFP	*RTG1::SAT1^R/RTG1::RTG1-GFP-HIS1*	This study
CaRtg3-GFP	*RTG3::SAT1^R/RTG3::RTG3-GFP-HIS1*	This study
ΔCartg1 CaRtg3-GFP	*RTG1::SAT1^R/RTG1::HIS1*<br>*ENO1::CaCAS9-SAT^R/ENO1*<br>*RTG3-GFP-URA3/ RTG3-GFP-URA3*	This study
ΔCartg3 CaRtg1-GFP	*RTG3::SAT1R/RTG3::HIS1*<br>*RTG1::URA3/RTG3-GFP-ARG4*	This study
ΔCaaep3 CaRtg3-GFP	*AEP3::CaSAT1^R/AEP3::HIS1*<br>*RTG3::URA3/RTG3-GFP-ARG4*	This study
ΔCaaep3 CaRtg1-GFP	*AEP3::CaSAT1^R/AEP3::HIS1*<br>*RTG1::URA3/RTG1-GFP-ARG4*	This study
ΔCappr13 CaRtg1-GFP	*PPR13::CaSAT1^R/PPR13::HIS1*<br>*RTG1::URA3/RTG1-GFP-ARG4*	This study

ΔCappr13 CaRtg3-GFP	*PPR13::CaSAT1^{R}/PPR13::HIS1*<br>*ENO1::CaCAS9-SAT^{R}/ENO1*<br>*RTG3-GFP-URA3/ RTG3-GFP-URA3*	This study
ΔCaaep3 ΔCartg1 CaRtg3-GFP	*AEP3::CaSAT1^{R}/AEP3::HIS1*<br>*RTG1::ARG4/ Δrtg1*<br>*ENO1::CaCAS9-SAT^{R}/ENO1*<br>*RTG3-GFP-URA3/ RTG3-GFP-URA3*	This study
ΔCaaep3 ΔCartg3 CaRtg1-GFP	*AEP3::CaSAT1^{R}/AEP3::HIS1*<br>*RTG3::ARG4/ Δrtg3*<br>*ENO1::CaCAS9-SAT^{R}/ENO1*<br>*RTG1-GFP-URA3/ RTG1-GFP-URA3*	This study
ΔCappr13 ΔCartg1 CaRtg3-GFP	*PPR13::CaSAT1^{R}/PPR13::HIS1*<br>*RTG1::ARG4/ Δrtg1*<br>*ENO1::CaCAS9-SAT^{R}/ENO1*<br>*RTG3-GFP-URA3/ RTG3-GFP-URA3*	This study
ΔCappr13 ΔCartg3 CaRtg1-GFP	*PPR13::CaSAT1^{R}/PPR13::HIS1*<br>*RTG3::ARG4/Δrtg3*<br>*ENO1::CaCAS9-SAT^{R}/ENO1*<br>*RTG1-GFP-URA3/ RTG1-GFP-URA3*	This study
CaRtg3-GFP CaRtg1-mCherry	*RTG3::SAT1R/RTG3::RTG3-GFP-HIS1*<br>*RTG1::RTG1-mCherry-ARG4*	This study
CaRtg3$_{Psites}$-GFP<br>*CaRtg1-mCherry*	*RTG3::SAT1^{R}/RTG3::rtg3$_{S231A,S235A,S247A,S251A,S398A,S401A}$-GFP-URA3*<br>*RTG1::RTG1-mCherry-ARG4*	This study
**Vector**	**Characteristics**	**Source**
pETMM41-MBP	*f1 ori, 6xHis-MBP, Kan^{R}, lacI*	Addgene
pETMM41-CaRTG1-MBP	*f1 ori, 6xHis-MBP-CaRTG1, Kan^{R}, lacI*	This study

pETMM41-CaRTG3-MBP	*f1 ori, 6xHis-MBP- CaRTG3, Kan^R, lacI*	This study
pFA-CaARG4	*ColE1 ori, CaARG4, bla (Amp^R)*	Walther *et* Wendland 2008
pFA-SAT1	*ColE1 ori, SAT^R (Streptotricin acetyltransferase) bla (Amp^R)*	Walther *et* Wendland 2008
pFA-CaHIS1	*ColE1 ori, CaHIS1, bla (Amp^R)*	Walther *et* Wendland 2008
pFA-CaURA3	*ColE1 ori, CaURA3, bla (Amp^R)*	Walther *et* Wendland 2008
pV1200	*f1 ori, $P_{T7,}$ CaCAS9, FRT-FLR NAT^R,*<br>*P_{SNR52}-sgRNA FRT, Amp^R*	Vyas *et al.* 2015
pV1200RTG1	*f1 ori, $P_{T7,}$ CaCAS9, FRT-FLR NAT^R,*<br>*P_{SNR52}-sgRNA-RTG1 FRT, Amp^R*	This study
pV1200RTG3	*f1 ori, $P_{T7,}$ CaCAS9, FRT-FLR NAT^R,*<br>*P_{SNR52}-sgRNA-RTG3 FRT, Amp^R*	This study
pAYCU257	*MglMH3.2R, bla (Amp^R)*	Defosse *et al.* 2018
pFA-yEmCherry-T_{TRP1}-CaARG4	*ColE1 ori, mCherry-T_{TRP1} CaARG4, bla (Amp^R)*	This study
pFA-RTG3-CaURA3	*ColE1 ori, RTG3-CaURA3, bla (Amp^R)*	This study
pFA-RTG3$_{Psites}$-CaURA3	*ColE1 ori, rtg3 $_{S231A,S235A,S247A,S251A,S398A,S401A}$ -CaURA3, bla (Amp^R)*	This study

* All strains used in this study were constructed on the basis of *BWP17* wild type strain. Only the introduced changes are indicated in the genotypes listed in the above table.

Supplementary Table S2 Oligonucleotides used in this study:

Primer name	Aim	5'-3' Sequence
**DB30**	Δ*Cartg3* construction	CACAATTGATTGCAATCACTTACAACAATCATTCTTAACAAAAACTAAATATGGAAGCTTCGTACGCTGCAGGTC
**DB31**	Δ*Cartg3, CaRTG-GFP* and *CaRTG3-HA* construction	CATAATAATAATACTTATACTTATACTACTACCTCCAGAAATTAAAATCGTAATCATCTGATATCATCGATGAATTCGAG
**DB32**	Verification of Δ*Cartg3*	GTATTAAGGCTCAACGAGGG
**DB33**	Verification of Δ*Cartg3*, *CaRTG3-GFP* and *CaRTG3-HA*	CTGAATGGTGCTGTTGATTC
**DB34**	Δ*Cartg3* construction	GTAACGCCAGGGTTTTCCCAGTCACGACGCAAGCAACGTGAGCTAAAACGATTGGAAGAAGAACAATAC
**DB35**	Δ*Cartg3* construction	GACCTGCAGCGTACGAAGCTTCCATATTTAGTTTTTGTTAAGAATGATTGTTGTAAGTGATTGCAATC
**DB36**	Δ*Cartg3* construction	CTCGAATTCATCGATGATATCAGATGATTACGATTTTAATTTCTGGAGGTAGTAGTATAAGTATAAG
**DB37**	Δ*Cartg3* construction	GCGGATAACAATTTCACACAGGAAACAGCCGAATAAACAGGATAGAGTTATTGAGCCATTAGTTGAAAC
**DB38**	Verification of ΔCartg3	GTTTGCGTTCAGATTACCAC
**DB39**	Verification of Δ*Cartg3*, *CaRTG3-GFP* and *CaRTG3-HA*	GGCACAGATTGAAGTTTTGG
**DB40**	Δ*Cartg1* construction	CTTATTTTTTACACAATTAGATAACTCAAACACACACTTGTAGTCATATTTAAAATGGAAGCTTCGTACGCTGCAGGTC
**DB41**	Δ*Cartg1, CaRTG1-GFP* and *CaRTG1-HA* construction	GAAAATAGAAAATAGAAAAAATAAACACTATAACAGTCGGTGAGTTTCTTTTTTATCTGATATCATCGATGAATTCGAG
**DB42**	verification of Δ*Cartg1*	GATACGCACACGACAATACATG
**DB43**	Verification of Δ*Cartg1, CaRTG1-GFP* and *CaRTG1-HA*	CCGAATGGCAAATACCAACC
**DB44**	Δ*Cartg1* construction	GTAACGCCAGGGTTTTCCCAGTCACGACGCTAACACAAATAAGAGAAGAAATATTTGTCCAGCATTTGTTTC
**DB45**	Δ*Cartg1* construction	GACCTGCAGCGTACGAAGCTTCCATTTTAAATATGACTACAAGTGTGTGTTTGAGTTATCTAATTGTG
**DB46**	Δ*Cartg1* construction	CTCGAATTCATCGATGATATCAGATAAAAAAGAAACTCACCGACTGTTATAGTGTTTATTTTTTC
**DB47**	Δ*Cartg1* construction	GCGGATAACAATTTCACACAGGAAACAGCGACATCAGATACTCATAAAAACTACCAATTTGATCCAGAG
**DB57**	Verification of Δ*Cartg1*	CTACCAATTCCTACTCCTGC
**DB48**	Verification of Δ*Cartg1*, *CaRTG1-GFP* and *CaRTG1-HA*	GATGCGGTCAAAAACTCTGG
**DB49**	*CaRTG3* reconstitution	CCGCC CTGCAG CAAGCAACGTGAGCTAAAACG
**DB50**	*CaRTG3* reconstitution	CCGCC GTCGAC AGAGAGAATGTGACCCTGTC

**DB51**	*CaRTG1* reconstitution	CCGCC CTGCAG GTCCAGCATTTGTTTCTGCC
**DB52**	*CaRTG1* reconstitution	CCGCC GTCGAC ATTCTGATCCGTACGGAGAG
**DB53**	Construction of *CaRTG3-GFP*	CCAACTACAATCCAGAAGATTTTTTTGCAGATATAGGTACTAGTACTGAGAATATCGGTGCTGGCGCAGGTGCTTC
**DB54**	Construction of *CaRTG1-GFP*	GTTAATCAAAAGTGCATCCACAAGTAAATCAGGTAGACGGGGAAGTACGAGTGGAGGTGCTGGCGCAGGTGCTTC
**DB55**	Verification of *CaRTG3* reconstitution	GATGAGCTCAAGTTCAAACC
**DB56**	Verification of *CaRTG1* reconstitution	CTGGATCGTGGCAAGAAAAC
**DB84**	Guide for CRISPR of Δ*Caaep3* for CaRTG1-GFP introduction	ATTTG CACAAGTAAATCAGGTAGAC G
**DB85**	Guide for CRISPR of Δ*Caaep3* for *CaRTG1-GFP* introduction	AAAAC GTCTACCTGATTTACTTGTG C
**DB87**	CRISPR editing of Δ*Caaep3* for *CaRTG1-GFP* introduction	AACGTGTTAATCAAAAGTGCATCCACAAGTAAATCAGGTAGACGAGGAAGTACGAGTGGA GGTGCTGGCGCAGGTGCTTC
**DB97**	Substitution of *CaAOX2* by *GFP* (reporter assay)	GTTTTTATATACTTTTTTTTTTATTAATTCGATATAGATTTATTCAATTACAAA ATG GGTGCTGGCGCAGGTGCTTC
**DB98**	Substitution of *CaAOX2* by *GFP* (reporter assay)	GACTATCTAACTTATATCAAACCATAAATATAAAGCAAACAAACAAACAAATCA TCTGATATCATCGATGAATTCGAG
**DB99**	Substitution of *CaAOX2* by *GFP* (reporter assay) and AOX2.1 probe amplification for EMSA	CCACTGAGCAAATTCCTACG
**DB100**	Substitution of *CaAOX2* by *GFP* (reporter assay)	CTCAACAAGTTTGGTCTCCTATTTC
**DB101**	*CaRTG3-HA* construction	CCAACTACAATCCAGAAGATTTTTTTGCAGATATAGGTACTAGTACTGAGAATATC GGATCCGGAATCTTTTACCC
**DB102**	*CaRTG1-HA* construction	GTTAATCAAAAGTGCATCCACAAGTAAATCAGGTAGACGGGGAAGTACGAGTGGA GGATCCGGAATCTTTTACCC
**DB105**	AOX2.4 probe amplification for EMSA FW	GTAAATCCATGACTCTCTTTTC
**DB106**	AOX2.1 probe amplification for EMSA RV	GAAGCAGTAAGCATTTTGTAATTG
**DB115B**	AOX2.2 probe amplification for EMSA FW	GCAAGTGACTCAATAATACTTCC
**DB116**	AOX2.2 probe amplification for EMSA RV	CGTAGGAATTTGCTCAGTGG
**DB117**	AOX2.3 probe amplification for EMSA FW	CCTTTCTTTCCTTTTTGAATTTC
**DB118**	AOX2.3 probe amplification for EMSA RV	GGAAGTATTATTGAGTCACTTGC
**DB119**	AOX2.4 probe amplification for EMSA RV	GAAATTCAAAAAGGAAAGAAAGG
**FP2-GFPRV**	Verification of GFP strains RV	CATAACCTTCGGGCATGGCACTC
**X2-CaURA3**	Verification of *CaURA3* integration	GTGTTACGAATCAATGGCACTACAGC

**X3-CaURA3**	Verification of *CaURA3* integration	GTGTTACGAATCAATGGCACTACAGC
**X2-CaHIS1**	Verification of *CaHIS1* integration	GGACGAATTGAAGAAAGCTGGTGCAACCG
**X3-CaHIS1**	Verification of *CaHIS1* integration	GGACGAATTGAAGAAAGCTGGTGCAACCG
**X2-SAT1**	Verification of *SAT1* integration	GCACACACTACTTAATATACACAGC
**X3-SAT2**	Verification of *SAT1* integration	GTGAAGTGTGAAGGGGGAG
**ARG4-FW**	Verification of *CaARG4* integration FW	GTACACGACCCACAGTTAGTC
**ARG4-RV**	Verification of *CaARG4* integration RV	GTACACGACCCACAGTTAGTC
**WK1**	Site-directed mutagenesis for *RTG3*$_{S231A;S235A}$ FW	AGAGGTGAACACCTAGCTGGATTGGGCGCTATCGCTGAGGATGAC
**WK2**	Site-directed mutagenesis for *RTG3*$_{S231A;S235A}$ RV	GTCATCCTCAGCGATAGCGCCCAATCCAGCTAGGTGTTCACCTCT
**WK3**	Site-directed mutagenesis for *RTG3*$_{S247A;S251A}$ FW	AATAATTTAGCTTTCGCCCCAGATTTCGCTAAACGACAAAGCATT
**WK4**	Site-directed mutagenesis for *RTG3*$_{S247A;S251A}$ RV	AATGCTTTGTCGTTTAGCGAAATCTGGGGCGAAAGCTAAATTATT
**WK5**	Site-directed mutagenesis for *RTG3*$_{S398A;S401A}$ FW	TTCCAAAGAAAGGGTGCTATTGAAGCTGGTGAATTGAGTGAA
**WK6**	Site-directed mutagenesis for *RTG3*$_{S398A;S401A}$ RV	TTCACTCAATTCACCAGCTTCAATAGCACCCTTTCTTTGGAA
**WK7**	*CaRTG3* amplification for cloning FW	CCGCCCTGCAGATGGGAGATTACTTAAACGAC
**WK8**	*CaRTG3* amplification for cloning RV	CCGCCCTGCAGGATATTCTCAGTACTAGTACC
**KL154**	Cloning of *CaRTG1* into *pMM41* FW	GGCGGGTCGACATGTCTCAATTCGGAGAGTTTTATGATACA
**KL155**	Cloning of *CaRTG1* into *pMM41* RV	CCGCCGCGGCCGCTTATCCACTCGTACTTCCCCGTCTACCTG
**KL156**	Cloning of *CaRTG3* into *pMM41* FW	CCGCCGTCGACATGGGAGATTACTTAAACGACAATTCAGC
**KL157**	Cloning of *CaRTG3* into *pMM41* RV	GGCGGGCGGCCGCTCAGATATTCTCAGTACTAGTACCTATAT
**KL158**	*CaRTG1* mutagenesis $_{34}$CTG$_{36}$ FW	GAGAGTTTTATGATACATATTCGGTTGAGAATAACCAACA
**KL159**	*CaRTG1* mutagenesis $_{34}$CTG$_{36}$ RV	TGTTGGTTATTCTCAACCGAATATGTATCATAAAACTCTC
**KL162**	*CaRTG*1 mutagenesis $_{202}$CTG$_{204}$ FW	ATGTGAAGAAGCAGTTATCATCGTCACTAAATACCGCAGG
**KL163**	*CaRTG*1 mutagenesis $_{202}$CTG$_{204}$ RV	CCTGCGGTATTTAGTGACGATGATAACTGCTTCTTCACAT
**KL164**	*CaRTG3* mutagenesis $_{190}$CTG$_{192}$ FW	CAGCTACATCAAAACCAATCGTTCAACATGAATTTTGACTC
**KL165**	*CaRTG3* mutagenesis $_{190}$CTG$_{192}$ RV	GAGTCAAAATTCATGTTGAACGATTGGTTTTGATGTAGCTG

**KL166**	*CaRTG3* mutagenesis $_{283}$CTG$_{285}$ FW	GTTACTCCGGCAGTGGTAATTCGAACGCAAACCTCAACAC
**KL167**	*CaRTG3* mutagenesis $_{283}$CTG$_{285}$ RV	GTGTTGAGGTTTGCGTTCGAATTACCACTGCCGGAGTAAC
**KL168**	*CaRTG3* mutagenesis $_{1396}$CTG$_{1398}$ FW	AATGGATCCAATACCTCGAGCTCGTCACAACCACAATCCC
**KL169**	*CaRTG3* mutagenesis $_{1396}$CTG$_{1398}$ RV	GGGATTGTGGTTGTGACGAGCTCGAGGTATTGGATCCATT
**KL173**	*CaRTG3* mutagenesis $_{130}$CTG$_{132}$ FW	GTAATAGTAACTTAGCCTCGCAACCTCAGCTAAATC
**KL174**	*CaRTG3* mutagenesis $_{130}$CTG$_{132}$ RV	GATTTAGCTGAGGTTGCGAGGCTAAGTTACTATTAC
**MD9**	*CaRTG1* tagging with mCherry FW	ATCCACAAGTAAATCAGGTAGACGGGGAAGTACGAGTGGAGGTGGTGTTTCAAAAGGTGAAGAAGATAAT
**MD10**	Generation of pFA-*yEmCherry*-Trp1ter-*CaARG4* vector FW	CCGCCCGTACGATGGTTTCAAAAGGTGAAGAAGATAATATGGCTATTAT
**MD11**	Generation of pFA-*yEmCherry*-Trp1ter-*CaARG4* vector RV	CCGCCGGATCCTTGGCTACTGAAGCCGCTACC
**MD12**	*CaRTG1* tagging with mCherry RV	TAGAAAAAAATAAACACTATAACAGTCGGTGAGTTTCTTTTTTAACTTAAGATTGATTTTAAATTTTCTAA
**MD15**	Amplification of MLS1.1 probe for EMSA FW	CGGAGATTTCCATGGTCGTG
**MD16**	Amplification of MLS1.1 probe for EMSA RV	GACATCAACTGTCGAAAAACCAAAC
**MD17**	Amplification of MLS1.2 probe for EMSA FW	CTAGCTTTGAAGTTGTCAAG
**MD18**	Amplification of MLS1.2 probe for EMSA RV	CGTCATAGTGTGACTACG
**MD19**	Amplification of MLS1.3 probe for EMSA FW	GTTGATGCACGAACATGACTTC
**MD20**	Amplification of MLS1.3 probe for EMSA RV	CTCGCCTGAACGGAAGTC
**MD21**	Amplification of MLS1.4 probe for EMSA FW	CTTCCGTTCAGGCGAGAG
**MD22**	Amplification of MLS1.4 probe for EMSA RV	GTTTCCAATTCACTAAAGGACCG
**MD23**	Amplification of NDE1.1 probe for EMSA FW	GATGAAGTTTTACTATGAACTGG
**MD24**	Amplification of NDE1.1 probe for EMSA RV	GGAACTTTGAAAGAAGTTCTTAG
**MD25**	Amplification of NDE1.2 probe for EMSA FW	CTTCTCCTTTCTTCCGTC
**MD26**	Amplification of NDE1.2 probe for EMSA RV	CATCCATATATCGTATTAGTGC
**MD27**	Amplification of DIC1.1 probe for EMSA FW	GACAAATTATTATAATTGGGTTAG
**MD28**	Amplification of DIC1.1 probe for EMSA RV	CATGTTAATCAAAAATACTCAAC
**MD29**	Amplification of DIC1.2 probe for EMSA FW	GCACCCAAAATAACTCCC

**MD30**	Amplification of DIC1.2 probe for EMSA RV	GCTACACTTTTGACAGCTG
**MD31**	Amplification of DIC1.3 probe for EMSA FW	CTCATCTACTATTCCCAGAG
**MD32**	Amplification of DIC1.3 probe for EMSA RV	CGCGATTTATCCTTCATAC
**MD33**	Amplification of DIC1.4 probe for EMSA FW	CACAATTAAGGCTTACTCAC
**MD34**	Amplification of DIC1.4 probe for EMSA RV	GTAATCTTCAAATGAGAGAGAG
**MD35**	Amplification of MCP1.1 probe for EMSA FW	CTCTTGTAGCAAATGCAC
**MD36**	Amplification of MCP1.1 probe for EMSA RV	CAATTTATTATTCAATGTTCATCG
**MD37**	Amplification of MCP1.2 probe for EMSA FW	GAGTCAATTGTTGTAATTTTG
**MD38**	Amplification of MCP1.2 probe for EMSA RV	CACAATCTTTAGATATAACACC
**MD39**	Amplification of MCP1.3 probe for EMSA FW	CTGCAGGATTTACATTCAG
**MD40**	Amplification of MCP1.3 probe for EMSA RV	GTTTATCAACACAAAATATCATTG
**MD41**	Amplification of PTC6.1 probe for EMSA FW	GTTGCAACTAGTTTATCAATC
**MD42**	Amplification of PTC6.1 probe for EMSA RV	CTAAATCGGATTTCGGTC
**MD43**	Amplification of PTC6.2 probe for EMSA FW	CAGCAATTGTTTATTAGATAGTG
**MD44**	Amplification of PTC6.2 probe for EMSA RV	GGACTTATACTTCCAGTTTG
**MD45**	Amplification of PTC6.3 probe for EMSA FW	CCTTATTGGTCGATGTAGTCC
**MD46**	Amplification of PTC6.3 probe for EMSA RV	GTTTGTGTGTATGTGTGCC
**MD47**	Amplification of CBP1.1 probe for EMSA FW	CCTAACACTTTAATTTAACAATAAC
**MD48**	Amplification of CBP1.1 probe for EMSA RV	CTCGTTTAGCTAGAACTAAC
**MD49**	Amplification of CBP1.2 probe for EMSA FW	CGTTATTCCCAGTCCAATC
**MD50**	Amplification of CBP1.2 probe for EMSA RV	GGTAACAAAACATCGTTTAACC
**MD51**	Amplification of CBP1.3 probe for EMSA FW	CGATAGGGAAACACACTAG
**MD52**	Amplification of CBP1.3 probe for EMSA RV	GTGAAACAACTATGTAGAATAAAAG
**MD53**	Amplification of CBP1.4 probe for EMSA FW	GATGAACGTAGAATTTGTTC
**MD54**	Amplification of CBP1.4 probe for EMSA RV	CAATCAACTATTACAAATTAACC

**MD55**	Amplification of MDM34.1 probe for EMSA FW	GCAAATTTGTTTAAGCTAGCTTG
**MD56**	Amplification of MDM34.1 probe for EMSA RV	CCTTCACGTCTGTTTTTTTTTTC
**MD57**	Amplification of MDM34.2 probe for EMSA FW	CATCAAGAAGAGAAATCAAGC
**MD58**	Amplification of MDM34.2 probe for EMSA RV	CTCATTAATTACTAATTCTATGCGG
**MD59**	Amplification of MDM34.3 probe for EMSA FW	CGACATTTACTTCTTCTTCTC
**MD60**	Amplification of MDM34.3 probe for EMSA RV	GGTGTGTGTTATAAGTCTTC
**MD61**	Amplification of MDM34.4 probe for EMSA FW	CCCTTGTTTTTGTAAATATTCTG
**MD62**	Amplification of MDM34.4 probe for EMSA RV	GCAAAAAGCTATAGGATTAGC
**MD63**	Amplification of PEX11.1 probe for EMSA FW	CATTAAGGGAGAAATTTGAAGC
**MD64**	Amplification of PEX11.1 probe for EMSA RV	CATCATCACCACCACTTC
**MD65**	Amplification of PEX11.2 probe for EMSA FW	GGATGAAATGAATGAACAAG
**MD66**	Amplification of PEX11.2 probe for EMSA RV	CAACCTTACTTTACCTTACC
**MD67**	Amplification of PEX11.3 probe for EMSA FW	CGTAATGCATTCATACATTC
**MD68**	Amplification of PEX11.3 probe for EMSA RV	GTGAAGTGGTTAAACACC
**MD69**	Amplification of PEX11.4 probe for EMSA FW	CACATACAAAACAAACTTCC
**MD70**	Amplification of PEX11.4 probe for EMSA RV	CAGCAAGTTAAGAAAGAAAG
**MD71**	Amplification of AXL2.1 probe for EMSA FW	GAGATCTACTTTAGTACTTTTG
**MD72**	Amplification of AXL2.1 probe for EMSA RV	GTTCAAAATAAACAATTGACTC
**MD73**	Amplification of AXL2.2 probe for EMSA FW	GATGCACACTTTATAGTTC
**MD74**	Amplification of AXL2.2 probe for EMSA RV	CAAAAGCTTAATAAAAATTTGAC
**MD75**	Amplification of AXL2.3 probe for EMSA FW	CCACAGACGACATAATTAC
**MD76**	Amplification of AXL2.3 probe for EMSA RV	CTCCGTCTCATTCTTATTG
**MD77**	Amplification of DDL1.1 probe for EMSA FW	GGCGACATACTGATATAC
**MD78**	Amplification of DDL1.1 probe for EMSA RV	CAAACTCTATGTTATTCCAG
**MD79**	Amplification of DDL1.2 probe for EMSA FW	CTACAATGTTTACACAAAATCC

**MD80**	Amplification of DDL1.2 probe for EMSA RV	CTCTTATTCATTTCTTATGTGC
**MD81**	Amplification of TRX1.1 probe for EMSA FW	CCACTGTTTGATTATTCGAAG
**MD82**	Amplification of TRX1.1 probe for EMSA RV	GTAAAAGAAACCTAGGAATCTG
**MD83**	Amplification of TRX1.2 probe for EMSA FW	GTTGATTTCTCAGCAACG
**MD84**	Amplification of TRX1.2 probe for EMSA RV	GGGGTGAATTACTAATATTCTTC
**MD85**	Amplification of DLD2.1 probe for EMSA FW	CCTGTGAATGTGAAAATAAAG
**MD86**	Amplification of DLD2.1 probe for EMSA RV	GAATTGGCAAAACTAACG
**MD87**	Amplification of DLD2.2 probe for EMSA FW	CCACTGGGATATCTAGTAATG
**MD88**	Amplification of DLD2.2 probe for EMSA RV	GCACATTCAATCCTATAACG
**MD89**	Amplification of DLD2.3 probe for EMSA FW	CACAATTTCGTTTTTATCAGC
**MD90**	Amplification of DLD2.3 probe for EMSA RV	CACACCGGAACATTTAAC
**MD91**	Amplification of DLD2.4 probe for EMSA FW	CTTTTATTCCGTACTTAGTTGG
**MD92**	Amplification of DLD2.4 probe for EMSA RV	CTCAGTTATGTGCATAATGAC
**mCherry-RV**	Verification of mCherry strains RV	GCAAATGGTAATGGACCACC

FW – forward, RV – reverse

Species	NCBI assembly ID
Agaricus bisporus	GCF_000300575.1
Allomyces macrogynus	GCA_000151295.1
Aspergillus nidulans	GCF_000011425.1
Batrachochytrium dendrobatidis	GCF_000203795.2
Botrytis cinerea	GCF_000143535.2
Caenorhabditis elegans	GCF_000002985.6
Capsaspora owczarzaki	GCF_000151315.2
Coccidioides immitis	GCF_000149335.2
Cryptococcus neoformans	GCF_000091045.1
Drosophila melanogaster	GCF_000001215.4
Fonticula alba	GCF_000388065.1
Fusarium graminearum	GCF_000240135.3
Homo sapiens	GCF_000001405.40
Linderina pennispora	GCF_002104995.1
Malassezia globosa	GCF_000181695.2
Mixia osmundae	GCF_000708205.1
Mucor lusitanicus	GCA_001638945.1
Neurospora crassa	GCF_000182925.2
Orbilia oligospora	GCF_000225545.1
Parastagonospora nodorum	GCF_000146915.1
Puccinia graminis	GCF_000149925.1
Rhodonia placenta	GCF_002117355.1
Rozella allomycis	GCA_000442015.1
Saitoella complicata	GCF_001661265.1
Schizosaccharomyces pombe	GCF_000002945.2
Scleroderma citrinum	GCA_046718565.1
Sclerotinia sclerotiorum	GCF_000146945.2
Ustilago maydis	GCF_000328475.2
Vairimorpha necatrix	GCF_036630325.1
Xylona heveae	GCF_001619985.1

Supplementary Table S3. NCBI Accession numbers for non-yeast genomes used as "outgroups" in the ortholog searches. The amino acid sequences for each species were downloaded using the datasets command (datasets download genome accession GCx_xxxxxxxxxxxx., --include protein) and converted to blast databases.
